# Ependymoma group-specific blood-brain barrier differences uncovered by a multi-omics approach

**DOI:** 10.1038/s41598-026-47499-2

**Published:** 2026-04-10

**Authors:** Julia K. Sundheimer, Julia Benzel, Aniello Federico, Stefanie Volz, Maximilian Knoll, Britta Statz, Tuyu Zheng, Szymon W. Kmiecik, Jürgen Burhenne, Gzona Bajraktari-Sylejmani, Sophia Scheuermann, Anke King, Torsten Müller, Jens-Martin Hübner, Mathias Kalxdorf, Heike Peterziel, Ina Oehme, Jeroen Krijgsveld, Christian M. Seitz, Marcel Kool, Stefan M. Pfister, Kristian W. Pajtler, Kendra K. Maaß

**Affiliations:** 1 Hopp Children’s Cancer Center Heidelberg (KiTZ), 69120 Heidelberg, Germany; 2 Division of Pediatric Neurooncology, German Cancer Consortium (DKTK), German Cancer Research Center (DKFZ), Im Neuenheimer Feld 580, 69120 Heidelberg, Germany; 3 Faculty of Biosciences, Heidelberg University, Im Neuenheimer Feld 234, 69120 Heidelberg, Germany; 4 Heidelberg Faculty of Medicine, Department of Pediatric Hematology, Oncology and Immunology, Heidelberg University Hospital, Im Neuenheimer Feld 430, 69120 Heidelberg, Germany; 5 National Center for Tumor Diseases (NCT), NCT Heidelberg, a partnership between DKFZ and Heidelberg University Hospital, 69120 Heidelberg, Germany; 6 Department of Radiation Oncology, Heidelberg University Hospital, Im Neuenheimer Feld 400, 69120 Heidelberg, Germany; 7 Clinical Cooperation Unit Radiation Oncology, German Cancer Consortium (DKTK), German Cancer Research Center (DKFZ), Im Neuenheimer Feld 280, 69120 Heidelberg, Germany; 8 Internal Medicine IX - Department of Clinical Pharmacology and Pharmacoepidemiology, Heidelberg University, Medical Faculty Heidelberg / Heidelberg University Hospital, Im Neuenheimer Feld 410, 69120 Heidelberg, Germany; 9 Clinical Cooperation Unit Pediatric Oncology, German Cancer Research Center (DKFZ), Heidelberg, Germany; 10 Department of General Pediatrics, Hematology and Oncology, University Children’s Hospital Tuebingen, Tübingen, Germany; 11 University of Tuebingen, iFIT Cluster of ExCellence (EXC2180) “Image-Guided and Functionally Instructed Tumor Therapies”, Tübingen, Germany; 12 German Cancer Research Consortium (DKTK), Partner Site Tuebingen, German Cancer Research Center (DKFZ), Heidelberg, Germany; 13 Division of Proteomics of Stem Cells and Cancer, German Cancer Research Center (DKFZ), Heidelberg, Germany; 14 Clinical Cooperation Unit Pediatric Oncology, German Cancer Research Center (DKFZ) and German Cancer Consortium (DKTK), 69120 Heidelberg, Germany; 15 Princess Máxima Center for Pediatric Oncology and University Medical Center Utrecht (UMCU), Utrecht, The Netherlands

**Keywords:** Blood-brain barrier, Tight junctions, Efflux pumps, Transporter, Receptor, Ependymoma, Cancer, Neuroscience, Oncology

## Abstract

A significant obstacle in treating brain tumors is the limited drug penetration across the blood-brain barrier (BBB), characterized by an interplay of endothelial tight junctions and efflux pumps. Brain tumors can alter BBB characteristics; however, there is limited understanding in ependymoma (EPN), the third most common pediatric brain tumor. To this end, we characterized EPN tumor (*n* = 364) and healthy brain tissues (*n* = 225) at RNA level and identified a distinct EPN group-specific BBB transcriptional pattern. Analyses of public datasets from Aubin and Gojo as well as a validation single-cell dataset (*n* = 8) could further specify a novel BBB signature expressed in an endothelial subpopulation. Clinically relevant drugs (*n* = 3) that were effective against EPN in vitro were further evaluated for BBB penetration in our subtype-specific patient-derived xenograft (PDX) models. Idasanutlin showed overall low brain-to-plasma ratios, while the P-glycoprotein (PGP) substrates temsirolimus and etoposide accumulated slightly more in zinc finger translocation associated (ZFTA)-fusion positive EPN than in PFA tumors and adjacent brain. These differences align with modestly lower PGP levels in ZFTA PDX, although expression does not necessarily reflect transporter activity and was not consistently observed in patient tumors. Despite these differences, all tested drugs remained below their effective in vitro levels. In summary, multi-omics analyses of BBB characteristics improve the understanding of drug penetrance and may potentially guide treatment choices in the context of molecular EPN groups within upcoming clinical trials.

**Supplementary Information:**

The online version contains supplementary material available at 10.1038/s41598-026-47499-2.

## Introduction

The physiological blood-brain barrier (BBB) is a non-fenestrated monolayer of tightly sealed endothelial cells forming the neurovascular unit together with astrocytes, pericytes, and other supporting cells. Tight junctions form complexes between those cells, including proteins such as occludin (OCLN), claudin family members, e.g. claudin-3 (CLDN3), claudin-5 (CLDN5), junctional adhesion molecules, and a number of cytoplasmic accessory proteins such as zonula occludens 1 (ZO1/TJP1), 2 (ZO2), and 3 (ZO3). Together, they restrict the paracellular transport, especially of large, hydrophilic compounds exceeding 800 Da^1,2^. In contrast, lipophilic drugs may passively diffuse through the cell membrane and cross the brain endothelium^1^. More specific BBB transfer mechanisms include solute carrier-mediated, receptor-mediated, and ion transport^1^. The maximum achievable concentration of many therapeutic compounds is further affected by ATP-binding cassette transporters actively pumping drugs back into the blood stream. The most important efflux pumps are P-glycoprotein (PGP, also known as ABCB1 transporter) and breast cancer resistance protein (BCRP, also known as ABCG2 transporter)^3,4^. Furthermore, the expression of BBB marker genes differs between different brain regions in human and mice with the greatest difference in BBB tightness between cerebellum and cortex and changes with age^5,6^.

Pathological processes, such as brain tumors may compromise the physiological BBB integrity. Brain tumors are among the leading causes of cancer-related morbidity and mortality, especially in children^7^. Therapeutic opportunities are limited, in part due to the restrictive characteristics of the BBB, which impede drug delivery to tumor cells. The development of effective therapies for intracranial tumors necessitates the integrative understanding of both tumor-specific vulnerabilities and BBB characteristics to achieve drug delivery and efficacy. Around 95% of drugs, that were preclinically found to be effective against brain tumor cells, cannot cross the BBB and therefore fail in clinical trials^8,9^.

In brain cancers, the BBB has been mainly studied in glioblastoma (GBM); here, high endothelial proliferation has been associated with widespread tumor infiltration and concomitant loss of tight junction expression in the tumor endothelium. In contrast, efflux pump expression remained stable or was even increased^10–12^. However, tumor entity-specific BBB alterations remain poorly understood for most other brain tumors. Nevertheless, BBB understanding is of particular interest in ependymoma (EPN), a group of difficult-to-treat and mostly chemoresistant brain tumors^13^.

EPN is molecularly subdivided into 10 distinct groups localized in the cortex, cerebellum, or spine^13^. Intracranial EPN comprise posterior fossa group A (PFA) and B (PFB), as well as sub-EPN (PF-SE). In the supratentorial region sub-EPN (ST-SE), the zinc finger translocation associated (ZFTA) fusion-driven, and yes-associated protein 1 (YAP1) fusion-driven groups occur. The PFA and ZFTA groups exhibit the worst outcome despite the standard of care involving resection and irradiation^13,14^. In other pediatric brain tumors, the clinical importance of disease-specific BBB characteristics was previously shown to influence outcomes. In the molecular WNT subgroup of medulloblastoma (MB), a highly fenestrated vascular system contributed to a favorable outcome^15,16^.

Correlative EPN studies such as BIOMECA have generated comprehensive molecular data for the identification of biomarkers, targetable alterations, and risk stratification^17^. Nevertheless, a methodology to translate molecular data into BBB characteristics is desirable.

To this end, our study systematically characterized BBB features across molecular groups of EPN, using a multi-omics approach. First, we investigated group-specific BBB differences by analyzing tumor bulk transcriptome data. Next, we allocated the expression of key BBB markers at the single-cell level in varying extents to endothelial cell populations. We further validated selected BBB-associated factors on the protein level. To evaluate the translational relevance of EPN mouse models, we assessed BBB characteristics in patient-derived xenograft (PDX) mouse models of ZFTA and PFA and an *in utero* electroporation (IUE) mouse model of ZFTA. Finally, we demonstrated the functional impact of observed BBB differences on drug penetration to both tumor and adjacent normal brain regions within PDX models. By elucidating BBB penetrance and efflux mechanisms, this study offers a resource for molecularly informed BBB characterization to guide effective drug delivery and improve outcomes of EPN patients by optimized trial designs.

## Results

### BBB expression patterns reveal EPN group-specific alterations

**Fig. 1:**
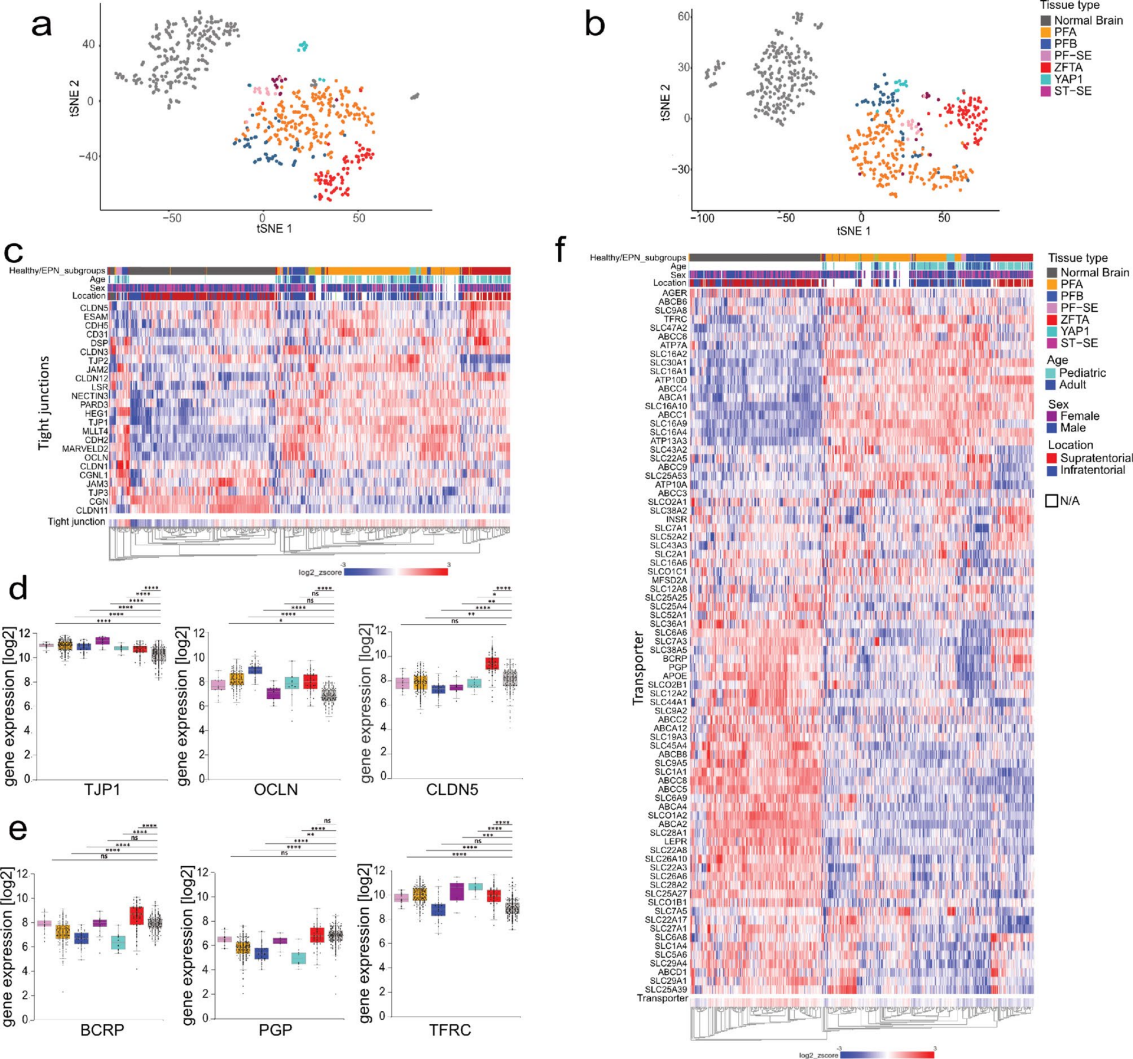
BBB-associated gene expression differs between EPN groups and healthy brain tissue. (**a**, **b**) tSNE plot based on the expression of (**a**) tight junction and (**b**) transporter and receptor gene sets. (**c**) Heatmap showing unsupervised hierarchical clustering of tight junction gene expression. (**d**, **e**) Boxplot for expression levels of (**d**) tight junction factors: TJP1, OCLN, and CLDN5, and (**e**) efflux pumps PGP, BCRP, and the receptor TFRC. Data shown as median, 25th and 75th quartile and whiskers extend to smallest and largest values within 1.5 times the distance between the quartiles. Outliers are plotted individually. (**f**) Heatmap showing unsupervised hierarchical clustering of transporter/receptor gene expression. Welch’s ANOVA test performed to calculate significance. Ns *P* > 0.05; *P < 0.05; **P < 0.01; ***P < 0.001; ****P < 0.0001.

Initially, we curated two distinct BBB-associated tight junction and transporter/receptor gene lists based on a comprehensive literature review^1,4,18–23^ (Table S1). On the basis of these lists, we analyzed Affymetrix RNA bulk gene expression datasets on the six human intracranial EPN groups (*n* = 364)^13^ and healthy brain tissues from various brain regions (e.g. cortex, cerebellum (*n* = 225)^24^). T-distributed stochastic neighbor embedding (tSNE) and unsupervised hierarchical clustering analyses based on these lists revealed clustering according to individual molecular EPN groups and healthy tissues (Fig. 1a–c, f). Unsupervised clustering of tight junction genes highlighted a distinct cluster, corresponding to ZFTA, characterized by elevated expression of various tight junction genes, including CLDN5 and desmoplakin (DSP). In contrast, CLDN5 expression was lower in other EPN groups compared to healthy tissue, while TJP1 and OCLN were consistently increased across tumors (Fig. 1d). Ingenuity Pathway Analysis identified transcription regulators such as snail family transcriptional repressor 1 (SNAI1) as a key upstream negative regulator of tight junction gene expression. Notably, low expression of SNAI1 led to high cadherin 5 (CDH5) and CLDN3 but low CLDN11 in ZFTA and PFA (Fig. S1a, b).

Hierarchical clustering using the transporter gene list revealed two main clusters with an overall higher expression of transporters in healthy brain samples than EPN tumors (Fig. 1f). BCRP was upregulated in ZFTA patients, whereas its expression was lower in PFB, PFA and YAP patients in comparison to healthy controls. The efflux pump PGP was significantly lower expressed in most EPN, except for ZFTA and PF-SE tumors (Fig. 1e, f), whereas the transferrin receptor (TFRC) receptor showed a significant upregulation in all EPN groups despite PFB (Fig. 1e, f). Transporter-focused upstream analysis revealed the ligand-dependent nuclear receptor estrogen receptor 1 (ESR1) as a key upstream regulator (Fig. S1c, d). However, ESR1 expression patterns did not consistently explain transporter expression across subgroups, suggesting involvement of alternative regulators such as activating transcription factor 4 (ATF4, (Fig. S1c, d)).

In summary, bulk transcriptomic analysis revealed markedly elevated expression of tight junction components across molecular EPN groups compared to healthy brain tissue. Expression patterns of efflux transporters showed no consistent patterns of deregulation.

### Developmental and regional BBB differences

**Fig. 2:**
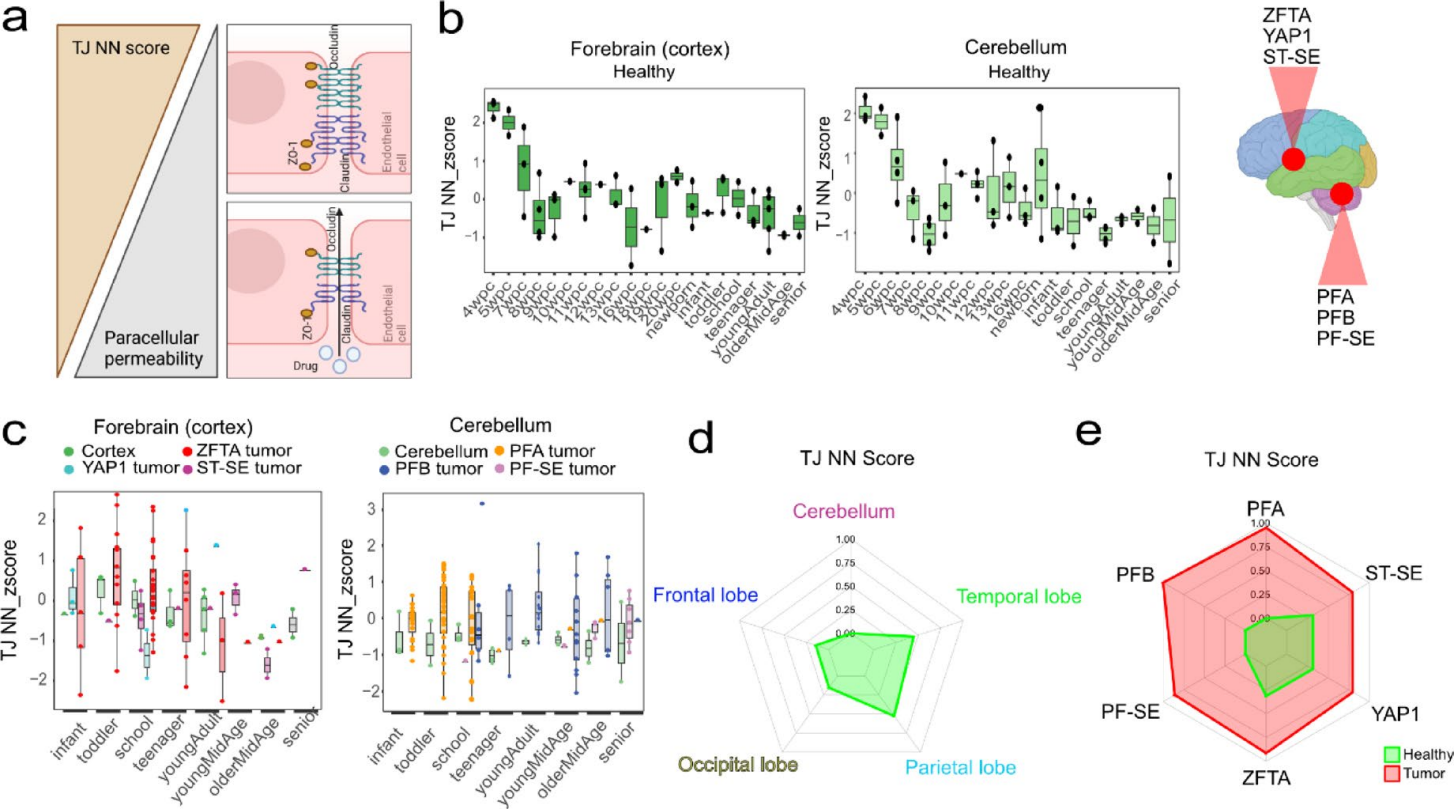
Age and brain-region dependent TJ NN scores differences do not reflect tumor phenotype. (**a**) Schematic representing translation of TJ NN score. (**b**) TJ NN score in healthy cortex or cerebellum at different development stages, (**c**) and in different EPN tumor tissues. Data shown as median, 25th and 75th quartile and whiskers extend to smallest and largest values within 1.5 times the distance between the quartiles. Data points outside this range are plotted individually as outliers. (**d**) Spider plots highlighting TJ NN score for healthy and (**e**) tumor samples.

To explore potential factors underlying EPN group-specific BBB expression patterns, we examined associations with patient age and tumor location within the human brain. To quantify these differences, we generated two distinct scores – one neural network-based tight junction score (TJ NN) and one neural network-based transporter score (TP NN) – applied to bulk transcriptomic data from various pediatric and adult brain tumors^25^. Feature importance analysis revealed that the TJ NN score was primarily driven by important junctional components including OCLN and TJP1 (Fig. S2a), indicating that higher TJ NN values reflect high endothelial barrier integrity and reduced paracellular permeability (Fig. 2a). In contrast the TP NN score was primarily driven by exporter SLC47A2, organic anion importer SLCO1C1 and lipid transporter APOE, whereas the efflux pump genes, PGP and BCRP, were ranked lower (9th and 12th, respectively), while TFRC did not contribute substantially (Fig. S2b). Thus, the TP NN score integrates importer and exporter systems and does not directly predict drug permeability, hence the clinical interpretation requires drug-specific consideration. In healthy cortex and cerebellum with well annotated ages and developmental stages^26^, the TJ NN score declined with increasing age, consistent with a more permeable BBB in older individuals (Fig. 2b)^6^. In contrast, the TP NN score showed a marked postnatal increase, particularly in the cortex, and continued to rise with age (Fig. S2c). However, these age dependent patterns were not observed in tumor samples (Fig. 2c, S2d). Although the higher TP NN score observed in PF-SE samples may partly reflect the fact that these specimens predominantly originate from older patients. Nevertheless, tumor TP NN score values were already elevated in the young-/mid-age groups compared to healthy cerebellum, without further increase in older age groups (Fig. S2d).

Brain region analysis revealed lowest TJ NN scores in the cerebellum compared to supratentorial regions (Fig. 2d). Importantly, TJ NN scores were higher in tumors and nearly identical across all EPN groups independent of brain region (Fig. 2e). Similarly, TP NN score was lowest in healthy cerebellum (Fig. S2e) and both posterior fossa and supratentorial tumors exhibited TP NN scores comparable to healthy cerebellum, independent of their anatomical origin (Fig. S2f). In summary, neither age nor the anatomical location had a strong influence on tumor-specific BBB characteristics.

## Single-cell transcriptomics reveals endothelial enrichment of BBB-associated genes

**Fig. 3:**
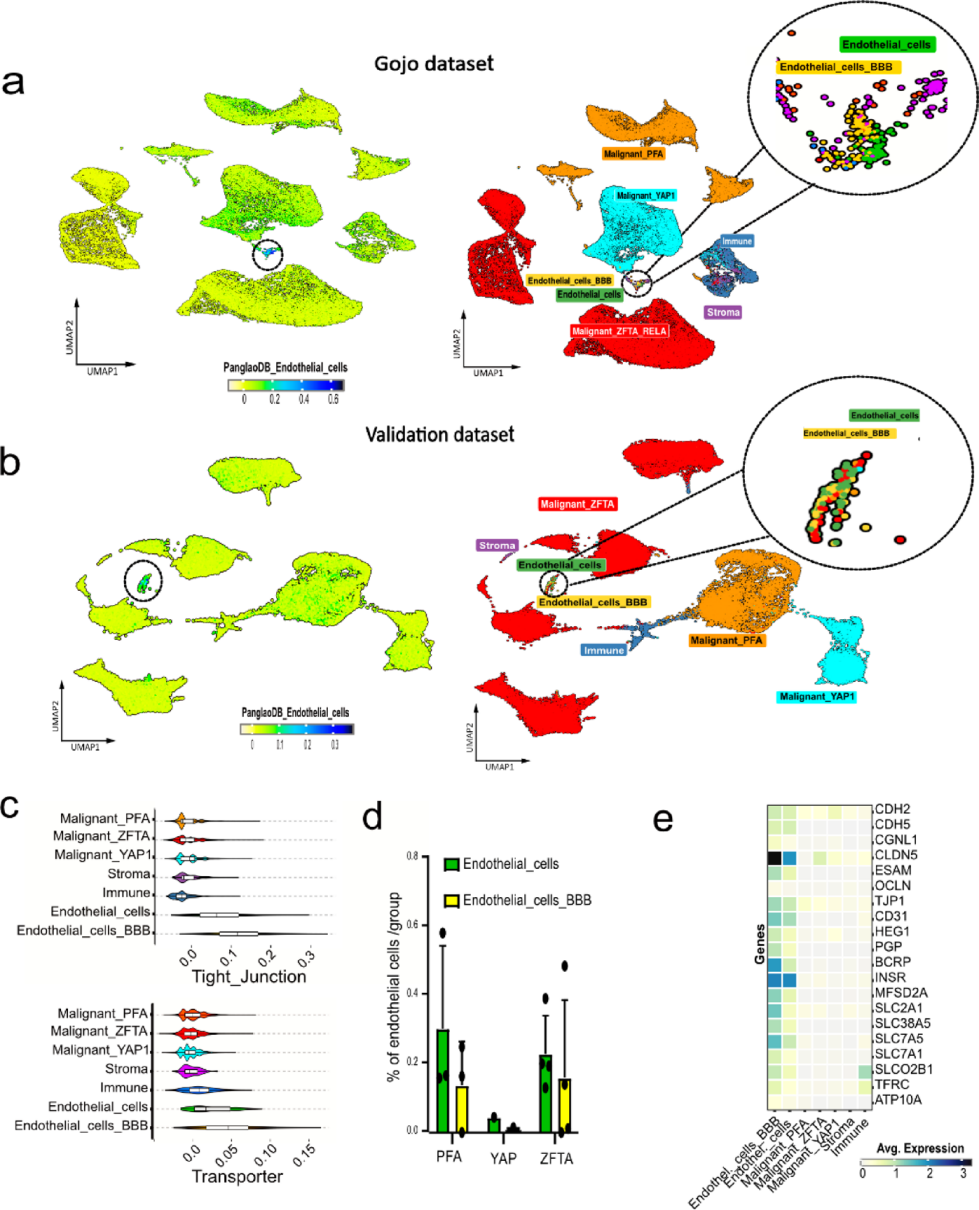
Single-cell analysis allocates main BBB-associated gene expression to endothelial cell clusters. (**a**) Uniform Manifold Approximation and Projection for Dimension Reduction (UMAP) with expression intensity of the Pangloa endothelial cell signature (left) and with annotated cell types of the Gojo dataset (right) and (**b**) of the validation single-nuclei dataset. (**c**) Violin plot of tight junction and transporter signature expression across all clusters (validation dataset). (**d**) Bar plots illustrating proportion of indicated endothelial cells in PFA, YAP1 and ZFTA molecular EPN groups. (**e**) Heatmap of the most differentially expressed tight junction and transporter genes in indicated single-nuclei clusters.

Given the limitation of tumor bulk analysis in resolving the cellular origins of observed expression patterns, we expanded the investigation of tight junction and transporter gene expression to human single-cell datasets. Following a comprehensive screening for published single-cell data, we selected sets from Gojo^27^ and Aubin^28^, as these cover the main pediatric EPN groups with a well-balanced amount of tumor, stroma, and endothelial cells. The “PanglaoDB_Endothelial_cells” gene signature, derived from the cell type marker database PanglaoDB (https://panglaodb.se/), allowed the identification of endothelial cells within the non-malignant cell clusters from the Gojo dataset (Fig. 3a). Among these, our defined BBB-specific tight junction and transporter gene sets further resolved this cluster into two distinct endothelial subpopulations suggesting the presence of a BBB-specialized endothelial phenotype. We annotated these subclusters as ‘endothelial cells_BBB’ – characterized by high expression of BBB-associated genes (yellow, Fig. 3a, Fig. S3c) – and ‘endothelial cells’, representing a population with lower BBB gene expression (green, Fig. 3a). In the Aubin dataset, both endothelial cell clusters were found but less prominent (Fig. S3h, i). To further validate these findings, we generated a new single-nuclei RNA sequencing dataset comprising all intracranial pediatric-type molecular EPN groups, including samples from four ZFTA, one YAP1, and three PFA patients. Both ‘endothelial cells_BBB’ and general endothelial cells populations were clearly observed (Fig. 3b). The BBB-associated genes were mainly expressed in ‘endothelial cells_BBB’, followed by general endothelial cells (Fig. 3c, Fig S3a). Tumor, stroma, and immune cells exhibited only minimal expression of tight junction and transporter genes in all datasets (Fig. 3c, Fig. S3d, i). These findings support endothelial cells as the principal source of BBB-related gene expression in EPN. Of note, the absolute number of cells within endothelial subpopulations for individual patients and proportions of subpopulations differed between molecular groups but also between datasets suggesting high heterogeneity between patients (Fig. 3d, Fig. S3e).

To evaluate whether endothelial phenotypes are conserved across brain tumor entities, we compared our endothelial subclusters with those identified in a recent GBM single-cell RNA-seq study^29^. Unlike GBM, the source of endothelial cells in EPN could not distinguished between tumor core and periphery. Nonetheless, the GBM-informed Pe1 (peripheral, BBB-enriched) and Co1 (core, angiogenesis-enriched) gene lists showed average expression patterns resembling with the ‘endothelial_BBB cluster’ across all three EPN datasets (Fig. S3b, f, j), suggesting partial conservation of BBB phenotypes between entities. Notably in our validation dataset, the GBM- informed Co2 (core, cytoskeletal-associated) gene list showed the highest correlation with the ‘endothelial_BBB cluster’ (Fig. S3b).

In contrast, the GBM clusters, Co3 and Pe2, with important roles described in immune cell recruitment, did not present a clear overlap with EPN endothelial cells (Fig. S3b, f, j), suggesting potential biological differences in vascular immune interaction between these tumor entities. While this may reflect distinct endothelial phenotypes, technical variation between single-cell technologies cannot be entirely excluded. Next, we investigated individual gene expression levels of tight junctions and transporters in all the newly identified cluster across all EPN datasets. Heatmaps indicating the top 20 upregulated genes highlighted consistent high expression of *CLDN5* in the endothelial_BBB cluster. These markers showed peak expression in endothelial_BBB cells, except for *INSR*, which was more prominent in the general endothelial cluster (Fig. 3e, Fig. S3g, k). Notably, key contributors (e.g. HEG1) to the TJ NN score were among the most upregulated genes at single-cell level, a pattern not observed for transporter genes (Fig. 3e, Fig. S2, S3g, k). These findings confirm that core BBB gene expression is confined to a specific endothelial subpopulation across multiple EPN datasets and can be extracted from bulk transcriptome data.

### RNA expression of tight junction factors predicts protein abundance in EPN

**Fig. 4:**
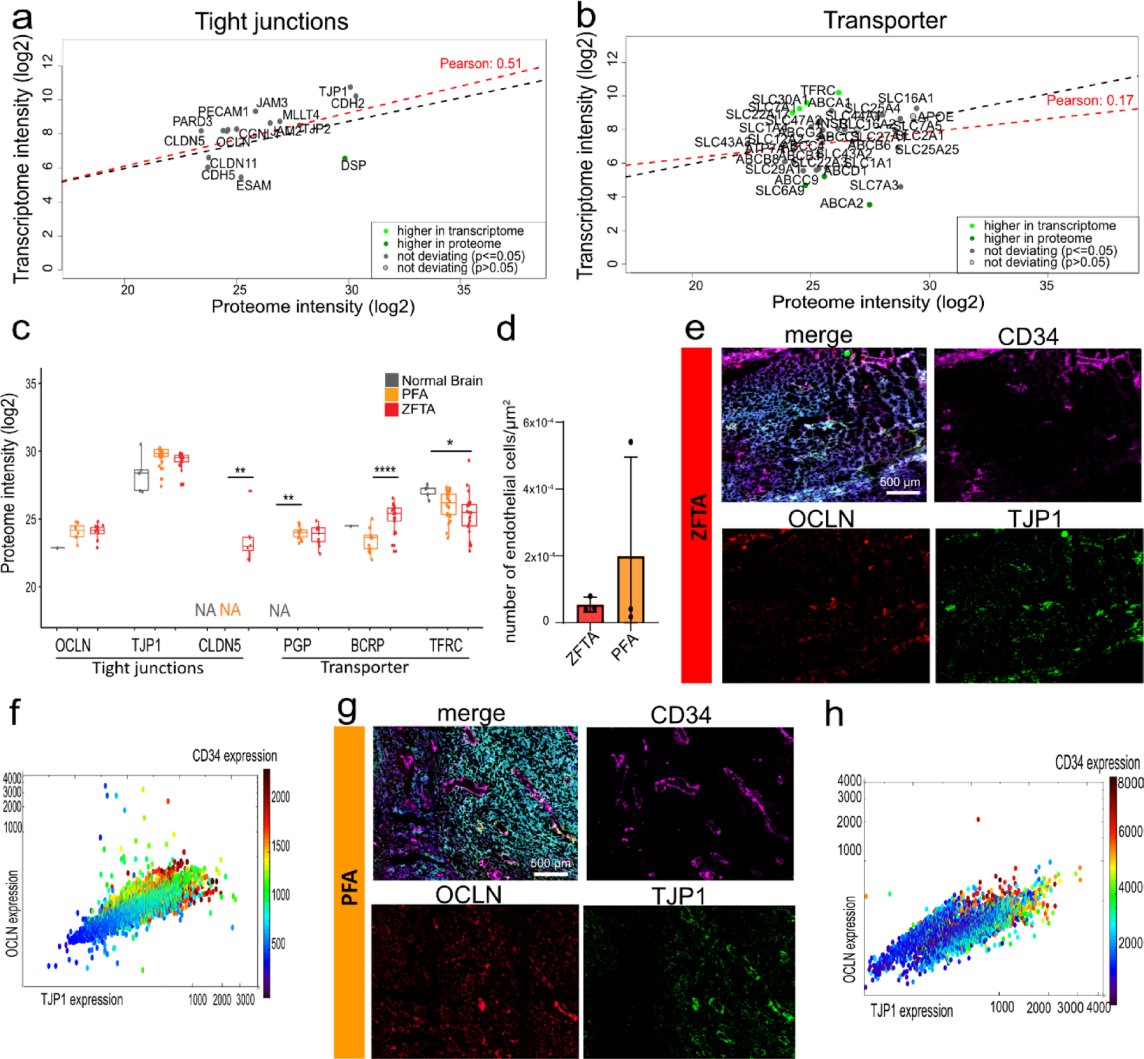
RNA-protein correlation and subcellular protein localization of tight junctions and transporters. (**a-b**) Linear regression model showing RNA to protein correlation of intracranial tumors for (**a**) tight junctions, and (**b**) transporter proteins. (**c**) Mass spectrometry-based protein levels of the tight junction factors OCLN, TJP1, the transporters PGP, BCRP and the receptor TFRC in ZFTA, PFA and healthy brain samples. (**d**) Quantification of endothelial cells based on CD34 in MACSima™ imaging cyclic staining (MICS). (**e**) MICS showing tumor cells (CD56, cyan) endothelial cells (CD34, pink), the transporters tight junctions OCLN (red) and TJP1 (green) and (**f**) the respective correlation of these three markers of a ZFTA patient and (**g**) PFA patient MICS and (**h**) correlation. Significance only indicated if significant *P < 0.05; ****P < 0.0001.

To evaluate the functional consequences of observed transcriptomic signatures on the protein level, we conducted mass spectrometry, supplemented by MACSima™ imaging cyclic staining (MICS) on human EPN samples. Of the defined tight junction gene set, 15 proteins were detected by mass spectrometry in all intracranial EPN, showing a higher correlation than for the global set (*r* = 0.51; Fig. 4a) exceeding that observed for transporter protein (*r* = 0.17, Fig. 4b) indicating tight junctions are more reliable reflecting proteomic levels. Group-specific analyses revealed similar trends for tight junctions (PFA: *r* = 0.43; ZFTA: *r* = 0.6; Fig. S4a, c) and transporter (PFA: *r* = 0.29, ZFTA: *r* = 0.29, FigS4b, d), though only fewer proteins were detected. Notably, CLDN5 expression exhibited higher transcriptome levels specifically in ZFTA tumors (Fig. S4a), nevertheless CLDN5 was enriched on protein level in ZFTA, but undetectable in healthy and PFA confirming its subtype-specific upregulation (Fig. 4c). Although TJP1 and OCLN were upregulated at the transcript level, their protein abundance was comparable across tumors and controls. In contrast to RNA levels, we observed that PGP levels were highly elevated at protein level in both tumor groups, while remaining below detection threshold for healthy brain tissues and TFRC protein levels were significantly decreased in ZFTA. BCRP levels reflected transcriptomic patterns (Fig. 4c). TSNE clustering of both tight junction and transporter protein expression revealed still separate cluster across EPN groups, but less distinct than in transcriptomic data, and interpretation is limited by the small number of samples (Fig. S4e, f).

To spatially confirm the endothelial localization of BBB-associated proteins, MICS was performed on patient EPN tumor tissue. First, we quantified the number of endothelial cells based on CD34 staining, revealing similar numbers for ZFTA and PFA patients, with higher variations between PFA patients (Fig. 4d). MICS technology confirmed endothelial-cell specific expression of TJP1, OCLN, PGP and BCRP (Fig. 4e–h, Fig. S4g) in tumor tissue stained with CD56 (cyan).

Overall, tight junction proteins, predominantly expressed in endothelial cells, show stronger RNA–protein correlation than transporters, suggesting better functional predictivity from molecular tumor data.

### PDX models recapitulate key BBB features for preclinical research

**Fig. 5:**
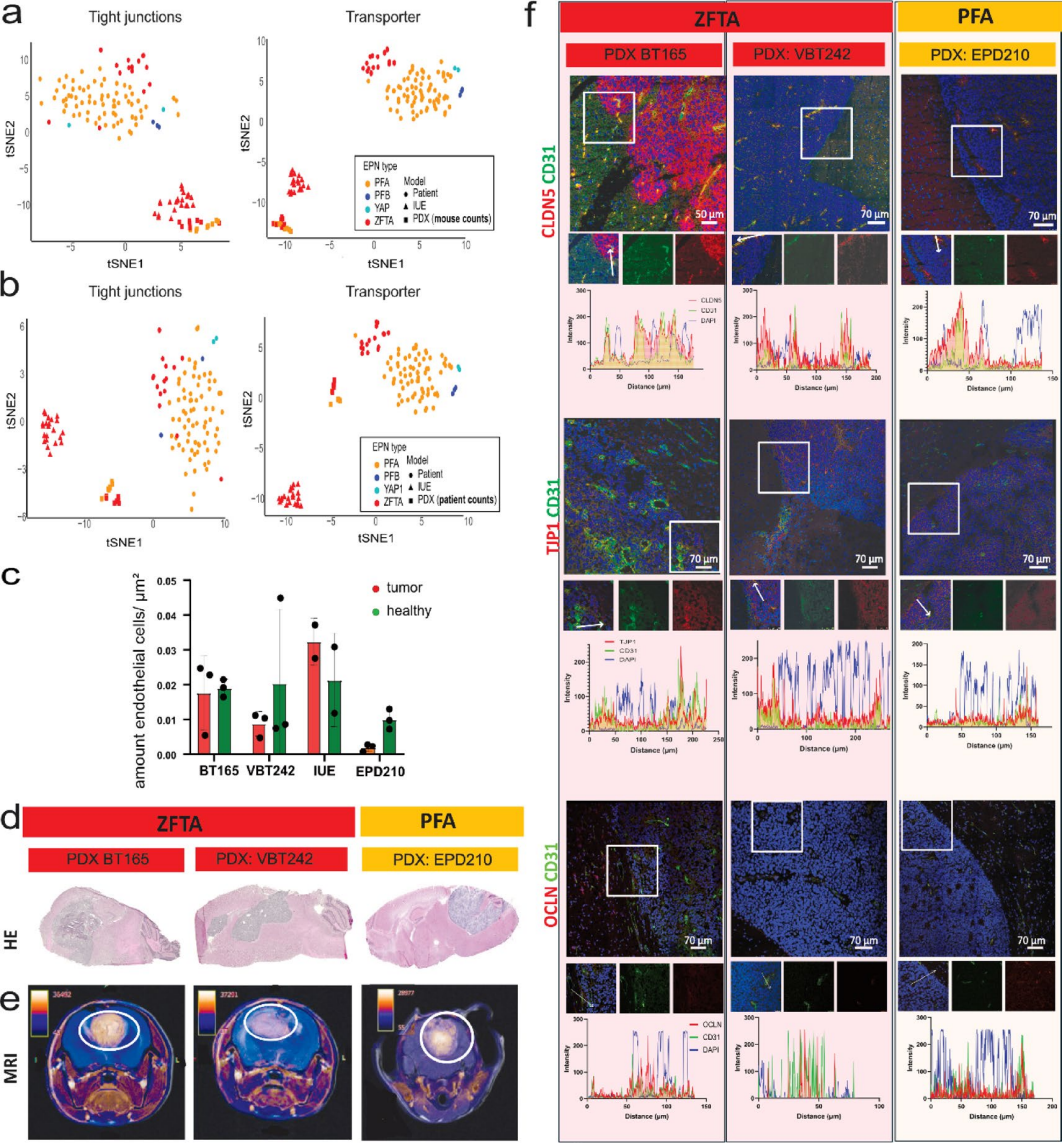
Tight junctions are highly expressed in endothelial cells of different mouse models. (**a**, **b**) TSNE of RNA sequencing data from patients, IUE, and PDX models using (**a**) mouse-specific counts and (**b**) human-specific counts for tight junction and transporter gene lists. (**c**) Quantification of endothelial cell density based on CD31 staining per area using CellProfiler, shown as mean ± SD. (**d**) H&E staining showing tumor expansion in different PDX models. (**e**) T2-weighted brain images (grayscale) overlaid with post-contrast T1-weighted images (color-coded, contrast agent enhancement intensity from low (purple) to high (orange)). (**f**) Immunofluorescence images using CD31 as endothelial marker co-stained with CLDN5, TJP1 or OCLN. Line plots below each image illustrate co-localization of CD31 with respective tight junction markers, confirming endothelial-specific expression. Images are representative and consistent across replicates.

Having confirmed the predictive value of molecular transcriptome data on the functional protein level, we next investigated the conservation of BBB characteristics between patients and mouse models being of importance for preclinical studies. We included both PDX (ZFTA- BT165, VBT242; PFA– EPD210) and IUE (ZFTA: ZFTA-RELA) models that molecularly recapitulate EPN tumors. In PDX models, endothelial cells are expected to be host-derived, which we confirmed by CD31 transcripts being mostly attributed to the mouse reference (Fig. S5a). However, most other BBB genes showed smaller proportions with mouse origin. Interestingly, the efflux pumps BCRP and at least one of the murine-equivalent isoforms of PGP revealed high murine proportions (Fig. S5a). Using newly generated RNA sequencing data from PDX and IUE mouse models combined with partly published cohort of patients^30^, tSNE clustering based on tight junction and transporter gene lists showed that mouse-specific counts separated patients from mouse models (Fig. 5a). In contrast, human-specific counts placed both ZFTA and PFA PDX models closer to patients, at least dependent on transporter gene list (Fig. 5b). The most notable difference between PDX and IUE models was an increased vascular diameter and higher endothelial cell density in IUE (Fig. 5c). Both ZFTA PDX models showed similar endothelial cell densities in tumor and adjacent healthy brain, whereas the PFA PDX model exhibited the lowest abundance of endothelial cells, potentially reflecting the variability observed in patients. Of note, the number of endothelial cells is not the only determinant of BBB permeability. As none of the models consistently mirrored patients, we focused on PDX models as these compromise both ZFTA and PFA. Tight junction protein levels in PDX models were validated by western blot analysis and revealed similar protein levels for TJP1 and OCLN in the ZFTA (VBT242) and PFA (EPD210) PDX tumors, consistent with previous observations in human tumors. In contrast, protein levels of both factors were lower in BT165 PDX tumor tissue, showing significance for OCLN suggesting patient-specific differences (Fig. S5d, e).

Although CLDN5 had been detected by mass spectrometry only in human ZFTA tumors, it showed strong protein expression in all PDX tumors relative to matched healthy tissue, reaching significance in PFA (Fig. S5c, d).

Further analysis of extratumoral regions revealed model-dependent expression differences within the same brain region (Fig. S5d, e). Specifically, TJP1 levels were elevated in ZFTA (BT165) cortex and cerebellum compared to both regions in the PFA model, whereas tumor-naïve mice tissue revealed similar protein expression as in ZFTA model (Fig. S5f), this suggests that tumor growth influences BBB alterations beyond the tumor core. Unlike human EPN, PDX tumors showed slightly lower PGP in ZFTA versus PFA and significant TFRC upregulation in VBT242 ZFTA tumor in contrast to healthy surrounding (Fig. S5c, d). Histological H&E assessment demonstrated overall low tumor infiltration across PDX models (Fig. 5d). For an initial assessment of BBB permeability, we studied contrast agent enrichment using gadolinium enhanced T1 overlaying T2-weighted MRI images in PDX models. Baseline (pre-contrast) T1 images were not available, thus, enhancement patterns are described qualitatively from post-contrast scans only. Both ZFTA models exhibited homogenous gadolinium enhancement throughout the tumor on post-contrast T1 images with more enrichment in the BT165 model (Fig. 5e). The PFA PDX model showed heterogenous enhancement with markedly hyperintense signal in tumor core on post-contrast T1 images (Fig. 5e). Localization of tight junctions and transporters was studied by immunofluorescence. All analyzed BBB markers (TJP1, CLDN5, OCLN, PGP, and BCRP) were restricted to endothelial cells across all PDX models (Fig. 5f, S5b, c). The only exception was CLDN5, which was also detected in tumor cells in BT165 ZFTA model, potentially influencing paracellular transport or indicating an alternative function of CLDN5 in tumor cells (Fig. 5f).

Overall, these results highlight the suitability of PDX models to study BBB penetration, as they capture patient-specific variability, including additional genetic alterations or bystander effects.

### Temsirolimus and etoposide penetration is higher in ZFTA tumor compared to healthy surrounding

**Fig. 6:**
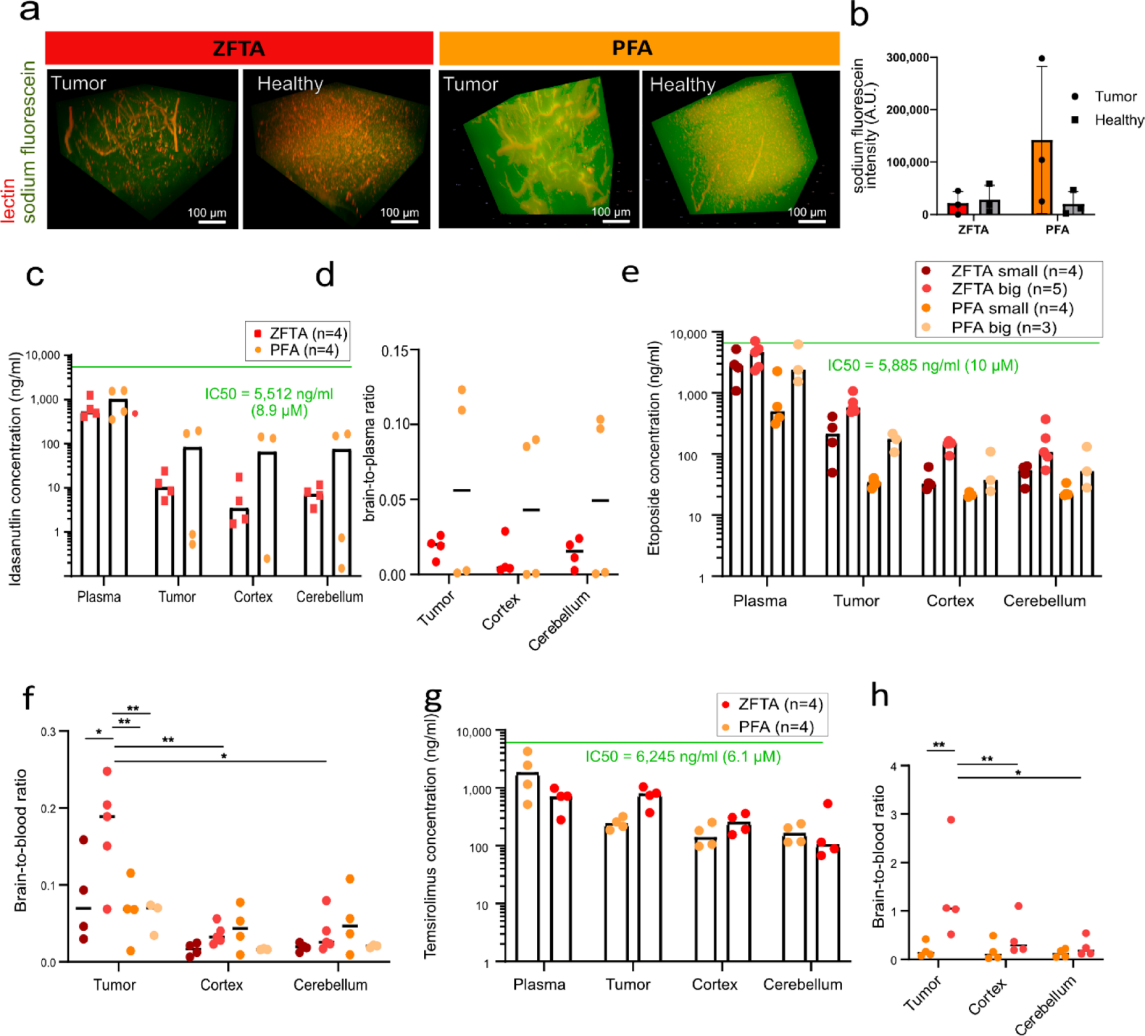
EPN group specific drug penetration in PDX models. (**a**) Sodium fluorescein (green) penetration relative to endothelial cells (lectin, red) in EPD210 (*n* = 3) and VBT242 (*n* = 3) PDX models, shown as 3D reconstructions of z-stacks and (**b**) corresponding quantification. (**c**) Idasanutlin concentrations in plasma and brain regions of a ZFTA (VBT242) and PFA (EPD210) PDX model following oral gavage (150 mg/kg) and (**d**) corresponding brain-to-plasma ratios. (**e**) Etoposide concentrations in small (< 100mm^3^) and large (> 150mm^3^) ZFTA (BT165) and PFA (EPD210) PDX tumors and brain regions after i.p injection (20 mg/kg) and (**f**) corresponding brain-to-plasma ratios. (**g**) Temsirolimus concentrations in plasma and brain regions of ZFTA (BT165) and PFA (EPD210) PDX models after i.p. injection (150 mg/kg) and (**h**) corresponding brain-to-plasma ratios. Statistical analyses were performed using repeated-measures one-way ANOVA (within models), ordinary one-way ANOVA (between tumors), and unpaired t-test for temsirolimus between tumor types. Ns *P* > 0.05; *P < 0.05; **P < 0.01.

Functional validation of BBB characteristics is essential to understand which drug classes display altered penetration in EPN compared to healthy brain. As a first step, we validated tight junction function by observing the penetration of sodium fluorescein, a small fluorescent tracer typically used to study BBB penetration. Based on this assay, ZFTA tumor tissue permeability was lower compared to the PFA tumor tissue (Fig. 6a, b).

Next, we investigated BBB penetration of the chemotherapeutic etoposide^31^ and the targeted inhibitors idasanutlin and temsirolimus. Drugs were selected based on an interdisciplinary decision process namely (i) in vitro response data (absolute IC50 values, Fig. S6); (ii) availability of validated UPLC-MS/MS methods pharmacokinetic measurements; and (iii) clinical translational potential. Both etoposide and idasanutlin are lipophilic with a molecular weight of around 600 Da, suggesting low likelihood of paracellular transport. Despite a moderate Gupta BBB score, idasanutlin showed a consistently low brain-to-plasma ratio of around 0.015 in ZFTA and 0,05 in PFA with no significant differences between tumor tissue and the corresponding cortex and cerebellum (Fig. 6c, d).

Etoposide, in contrast, is a known substrate of PGP and other multidrug resistance transporters such as ABCC2 and ABCC6^32^. We therefore analyzed etoposide penetration in both PFA and ZFTA tumors, also taking tumor volume into account. Although PGP expression did not differ significantly, a trend toward lower levels was observed in ZFTA tumor tissue, particularly in both ZFTA PDX mouse models (Figs. 4e and 5d). Strikingly, etoposide penetration was significantly higher in large ZFTA tumors, with a brain-to-plasma ratio approaching 0.2, compared to corresponding healthy brain regions as well as small ZFTA and PFA tumor (Fig. 6e, f).

Temsirolimus, a larger molecule, unlikely to undergo paracellular transport, is known as a PGP and transferrin substrate, suggesting a potential contribution of TFRC to its transport. Remarkably, temsirolimus concentrations and brain-to-plasma ratios were significantly higher in ZFTA compared to PFA tumors and healthy brain tissue (Fig. 6g, h), despite only slightly lower PGP and comparable TFRC expression in the BT165 ZFTA model relative to PFA PDX models. This finding suggests that even subtle reductions in PGP expression may enhance temsirolimus penetration (Fig. 5d). In summary, sodium fluorescein penetration was limited in the ZFTA group, indicating reduced paracellular permeability aligning with elevated tight junction levels in ZFTA patients (Fig. 1c), although sodium fluorescein transport may also depend on compound-specific characteristics. Regarding drug permeability, idasanutlin showed no difference in accumulation between tumor and adjacent healthy brain, whereas temsirolimus and etoposide (in large tumors) exhibited higher accumulation compared to healthy regions. Since both drugs are known PGP substrates, their enhanced permeability might be linked to reduced PGP expression in ZFTA PDX models. However, transporter expression levels do not necessarily reflect functional transporter activity, and caution is warranted when extrapolating these findings to patients. Indeed, PGP differences between PDX subgroups do not fully mirror human tumor expression patterns, underscoring the need for functional validation and careful consideration before therapeutic translation.

## Discussion

One of the major hurdles in identifying effective drugs for pediatric neuro-oncology is the limited ability to predict their BBB penetration^33^. Knowledge of differences between the BBB in healthy and tumor tissues is, therefore, highly relevant. In this study, we generated a multi-omics dataset of EPN patients and mouse models, revealing EPN group-specific differences in BBB-associated genes compared to normal tissue. While the BBB is generally believed to be disrupted in brain tumors^10,11^, we observed increased tight junction expression across intracranial EPN, suggesting reduced paracellular permeability. In contrast, no consistent trend was observed for transporters and receptors, which instead exhibited group-specific alterations.

Our established TJ and TP NN scores reflected known brain region-specific differences^34,35^, but also indicated region-independent changes in EPN. The age-associated decrease in BBB transporters was not mirrored for PGP and BCRP in the literature, as PGP levels increase from the perinatal to adult stage, whereas BCRP expression remains largely stable throughout development^36–39^. Since some tight junction and transporter genes are also implicated in oncogene regulation and apoptotic signaling^40,41^, we validated their primary localization in endothelial cells. Our single-nuclei dataset, together with re-analysis of published single-cell studies, confirmed their localization in endothelial cells and revealed a previously uncharacterized endothelial subcluster in EPN, called “endothelial_BBB cluster”. This cluster strongly correlated with two endothelial cell populations previously described in GBM, namely a peripheral cluster enriched for BBB markers (Pe1) and a core-associated angiogenic cluster (Co1)^29^. However, a comparison between tumor core and periphery in EPN remains limited due to lack of spatially resolved datasets.

To address RNA-protein translation, we calculated RNA-protein correlations, which were consistent with findings in medulloblastoma^42^. Although comparisons across datasets are methodologically challenging^43^, we observed positive correlations for most tight junctions and transporters. Exemplary staining confirmed their endothelial localization, supporting the utility of RNA-based TJ NN and TP NN scores to estimate patient-specific BBB permeability. However, interpretation of transporter gene expression is substrate-specific, since drugs rely on distinct transporters and TP NN score includes both import and efflux transporters.

We further compared BBB features across mouse models, which remain essential tools in preclinical drug testing. Both IUE and PDX models clustered apart from patient samples. A key distinction was the higher abundance of endothelial cells in IUE ZFTA models compared to ZFTA PDX models. This may reflect differences in age (PDX: 5–6 months; IUE: 2 month), tumor induction method, or genetic background (NSG for PDX/ CD1 for IUE)^39,44,45^. Age-associated BBB differences are conserved across humans and rodents, with protective functions declining with age^44^. Since electroporation for IUE induction occurs during embryonic barrier formation (E10–E15), it may also affect BBB physiology^4,46^.

A more detailed analysis revealed elevated CLDN5 RNA and protein levels in ZFTA patients. In PDX models, CLDN5 was highly expressed in both PFA and ZFTA tumors relative to healthy brain but uniquely enriched in both tumor cells and endothelial cells in the ZFTA BT165 model. This suggests a role beyond endothelial tight junctions, consistent with findings in glioma cell lines where lower CLDN5 expression correlated with higher tumor grade^47^. Whether a similar link exists to EPN aggressiveness remains to be tested. Functionally, low endothelial CLDN5 expression has been linked with increased permeability for smaller molecules (< 800 Da)^48^, consistent with our finding of reduced sodium fluorescein penetration in ZFTA tumors.

Other tight junctions, including OCLN and TJP1, were upregulated in ZFTA and PFA patient samples compared to healthy brain, but in mouse models this pattern was only confirmed for TJP1 in VBT242 (ZFTA) and EPD210 (PFA). SNAI1, a known repressor of tight junctions, was expressed in EPN patients, in line with literature linking it to BBB disruption and epithelial to mesenchymal cell transition^49,50^.

MRI-based assessment further supported our molecular findings. Gadolinium-enhanced T1 MRI revealed variability between ZFTA and PFA PDX models. However, interpretation was limited by missing baseline T1 images. Patient MRI data also showed variability, but nonetheless, recent advances in radiomic signatures already enable image-based differentiation of supratentorial EPN from other brain tumor entities, and future approaches may further refine EPN subtype^51^. While T1-weighted imaging indicated BBB disruption, dynamic-contrast enhanced MRI offers higher sensitivity^52^. Indeed, a recent study including posterior fossa EPN patients reported higher BBB penetration compared to medulloblastoma^53^, although direct comparison between ZFTA and PFA remains unresolved.

Drug penetration analysis revealed distinct subgroup-specific differences in EPN that were independent of anatomical brain region. Although the mechanisms of BBB transport remain incompletely understood for many agents, most are substrates of PGP and/or BCRP^54^, and importantly, transporter expression levels do not necessarily correlate with functional activity^57^. Idasanutlin, predicted to have moderate BBB penetration based on the Gupta score, showed consistently low brain-to-plasma ratios in both ZFTA (~ 0.02), PFA (~ 0.05) tumors and healthy brain, contrasting with higher ratios reported in CD1 mice^55^, likely due to strain-specific differences, and suggesting limited tumor-specific accumulation via predominantly transcellular transport. Etoposide, predicted to have low penetration, reached brain-to-plasma ratios of approximately 0.1 and accumulated more strongly in large ZFTA tumors compared to PFA and healthy tissue, consistent with slightly reduced PGP protein levels in ZFTA PDX tumors. However, as etoposide is also a substrate of ABCC1/2/3/6 ^32,56^ and patient RNA data revealed higher ABCC6 expression in PFA, multiple efflux mechanisms likely contribute to subgroup differences. Temsirolimus, also predicted to have low BBB penetration and known to be a PGP substrate, accumulated in ZFTA but not PFA tumors despite only modest differences in PGP expression, again underscoring that expression does not necessarily reflect activity^57^. Although temsirolimus binds transferrin, the ability of the complex to engage TFRC^58,59^ remains unclear; TFRC protein levels were comparable in PDX models but reduced in ZFTA patient samples, suggesting a limited role in mediating tumor penetration. To enhance temsirolimus delivery and better define the contribution of PGP and TFRC, combination strategies with respective inhibitors could be considered. Overall, paracellular transport appears limited, particularly in ZFTA tumors, and drug penetration depends strongly on functional transporter activity. Future integrative analyses combining matched molecular profiling and pharmacokinetic data from patients will be critical to directly correlate tumor drug exposure with tumor response as well as clinical outcome to enable more precise subgroup-specific therapeutic strategies.

In conclusion, molecular EPN groups harbor different BBB characteristics independent of brain region and age. Overall, our data demonstrate that EPN maintain a more intact BBB than is typically observed in other brain tumors, with group-specific features that may inform therapy selection.

## Method details

### Human tissue processing

All experiments in this study involving human tissue or data were conducted in accordance with the Declaration of Helsinki and approved by the ethics committee of the Medical Faculty of Heidelberg University (S-531/2020, S-502/2013; S-795/2020). Written informed consent was obtained from all participants or their legal guardians prior to inclusion in the study. Global BBB gene expression in EPN patients and healthy controls (Table S3) was analyzed in Affymetrix dataset from a combined EPN tumor cohort with integration of normal brain tissues available on R2 platform (http://r2.amc.nl)^13,24^. Bulk RNA Affymetrix datasets^13,60,61^ were analyzed using the paired samples Wilcoxon test in the statistical software R.

### RNA isolation

Tissue homogenates were processed to isolate RNA by following the manufacture´s protocol of the Maxwell^®^ RSC simply RNA Tissue Kit (Promega, USA). Total RNA sequencing libraries were generated with TruSeq RNA library Prep kit (Illumina, USA) followed by RNA sequencing using NextSeq550/Novaseq S4 with PE 100 (Illumina, USA) in the Genomics Core Facility of the DKFZ (Heidelberg, Germany).

### TJ and TP score calculation

The TJ NN and TP NN scores were calculated from log2 expression values of all tight junctions and transporters from manually curated gene list (Table S1) by training a neural network with an 80% training set of a comprehensive dataset (*n* = 2646). This dataset includes samples from different brain tumors including adults and children covering various brain regions. The score was applied to EPN patients, healthy controls and healthy controls from different developmental stages as indicated in Table S3^13,24,26^. Spider plots were created with the radarchart function in R package fmsb.

### Single-cell and single nuclei sequencing

#### Sample processing and nuclei isolation

Nuclei were isolated from snap frozen tumor samples (*n* = 8) as previously described^62,63^. Briefly, tissue samples were fragmented in “CHAPS, with salts and Tris” (CST) buffer on ice. Cell debris was removed by filtering through 40 μm cell strainers (Greiner, Germany). After centrifugation, nuclei were counted using the Luna Automated cell counter (Logos Biosystems, South Korea) and approximately 9000 nuclei per sample were loaded onto the Chromium single-cell 3′ chip (10X Genomics, USA).

#### 10X single-nuclei library preparation and sequencing

The Chromium Next GEM Single-Cell 3ʹ Reagent Kits v3.1 (10X Genomics, USA) was used to prepare single-nuclei gene expression libraries according to the manufacturer’s instructions. RNA reverse transcription was performed in separate GEMs (Gel Bead-In-Emulsion) and on separate nuclei. Next, amplified and size-selected enriched cDNA formed the gene expression libraries and were analyzed for quality and quantity using the Qubit dsDNA HS Assay Kit (ThermoFisher Scientific, USA) and TapeStation (Agilent, USA). Equimolar pooled libraries (multiplexes) were sequenced on a NovaSeq 6000 (Illumina, USA) sequencer using S4 flow cell with paired-end reads, according to the manufacturer’s instructions, targeting approximately 50,000 reads per nucleus.

#### Data processing and downstream analyses

After pre-processing of raw sequencing data (FASTQ format) with the Cell Ranger pipeline (v6.1.1, 10X Genomics), reads were aligned to the GRCh38 human genome reference to identify expressed genes. Gene expression levels were assessed based on Unique Molecular Identifiers (UMIs) or individual transcript molecules. All downstream analyses described below were applied to this new validation dataset and publicly available single-cell and single-nuclei RNAseq datasets^27–29^ for cross-validation as indicated in Table S3. Expression count matrices were generated by using the Seurat package (v3.23) within R (v4.0.1). To ensure high-quality data for downstream analysis, filtering steps were applied to each dataset, as described previously^64^. Data were normalized and scaled using the SCTransform function in Seurat, followed by principal component analysis (PCA) and Uniform Manifold Approximation and Projection (UMAP) for visualization and clustering purposes. These methods allowed the identification of distinct cellular clusters representing various cell types present in each sample. To limit sample-specific technical variations, Harmony (v1.0;^65^) was employed for integration. Cell type annotations were determined by analyzing gene markers for each cluster using Seurat’s FindAllMarkers function. The endothelial_BBB cell population was identified through the expression of canonical endothelial markers and the presence of tight junction and transporter gene signatures, as described in this study and corroborated by findings from Xie et al.^29^. Gene expression signatures were scored using Seurat’s “AddModuleScore” function, generating module scores per cell that were compared across clusters and EPN groups. Data visualizations were generated using the Seurat and SCpubr packages (v2.0.2;^66^).

### Mass spectrometry

Peptides of lysed EPN tissue samples (overview in Table S3) were separated by an Easy NanoLC 1200 system with various C18 analytical columns (Acclaim PepMap RSLC, self-packed Reprosil-Pur, nanoEase BEH C18) coupled to Orbitrap Fusion or Q-Exactive HF instruments (Thermo Fisher Scientific). MS1 scans were acquired at 60,000 resolution (m/z 350–1500), with MS2 scans in DDA mode at 15,000 resolution using stepped collision energy.

Raw data were analyzed with MaxQuant (v1.5.1.2) against the human Uniprot database using trypsin/P digestion, carbamidomethylation (C) as fixed, and oxidation (M)/acetylation (N-term) as variable modifications. Label-free quantification (LFQ) (provided in Table S2), iBAQ, and matching between runs were enabled. Data processing was done in Excel, Perseus (v1.6.1.3), and R (v3.5.1).

### RNA-protein correlation

Proteome data was pre-processed by normalizing and checking for batch effects. Median protein and RNA transcript intensities of matched samples were used to analyze RNA-to-protein correlation and a mean linear regression model was calculated over all genes. Residuals were calculated by predicting transcriptome intensities from proteome intensities using the above fitted general linear model and finally subtracting observed transcriptome intensities from predicted intensities for every gene and every sample individually. Differential correlated genes were identified by using the lmFit function of the limma R package. We filtered for significance based on a log-fold change (logFC) greater than or equal to 2 and an adjusted p-value (adj.Pval) less than 0.05 Barplot.

### MACSima™ imaging cyclic staining (MICS)

Human EPN tumor samples were cut in 4 μm tissue slices using a cryotome (Leica CM1950). Tissue slides were fixed in 4% PFA for 10 min and antibody staining was performed as previously described^67^. Our antibody panel was adapted from Scheuermann et al.^67^ with the following antibodies (Table 1) and correlation analysis was performed using the MACS iQ View software (version 1.3.1, Miltenyi Biotec, Germany).

**Table 1:** Antibodies added to the established MACSima^TM^ panel.

Antibody	Clone	Order number	Dilution	Fluorochrome
ABCG2 (BCRP)	REA909	130-115-328	1:50	PE
CD338 (BCRP)	5D3	12-8888-42	1:50	PE
Mdr1 (PGP)	D11	sc-55510	1:50	FITC
OCLN	E5	sc-133256	1:50	FITC
TJP1	NBP2-99094	NBP2-99094 F	1:50	FITC
ZO1 (TJP1)	1A12	33-9111	1:50	FITC

### H&E and immunofluorescence staining

The tissue was sectioned with the Leica CM1950 with a thickness of 10 μm for H&E staining and immunofluorescence staining. For H&E staining, tissue slides were fixed for 30 min in 4% PFA, washed in water and stained in haematoxylin (2:45 min). After washing rinsing 1 min with water, dipping in 100% ethanol and washing again 4 min with water, slides were incubated 30 s in 95% ethanol prior to staining with eosine for 30 s. Then tissue slides were dehydrated in ascending ethanol solutions (70%, 95%,100%) and cleared with xylene for 2 min. Tissue was mounted with Eukitt (Orsatec, Germany). For immunofluorescence, sections were fixed in 4% PFA for 10 min and washed 10 min with PBS. Antigen retrieval was achieved by boiling 30 min in citrate buffer (10 mM sodium citrate tribasic dihydrate and 0.05% Tween20 in ddH2O; pH = 6.0), followed by 20 min cooling in this buffer. Cells were permeabilized with PBS with 0.1% Triton-X and blocked in 3% BSA in PBS for 1 h at RT. Primary antibodies were incubated at 4 °C overnight, in the dilutions indicated in Table 2. After 3 washing steps in PBS for 10 min, the secondary fluorophore-coupled antibodies were added in 3% BSA in PBS for 1 h at RT prior to mounting in DAPI-fluoromount. Only TJP1 and CD31 co-staining was performed without antigen retrieval step. Imaging was performed at confocal SP8 microscope (Leica, Germany).

**Table 2:** Overview of used antibodies in western blot and immunofluorescence.

Antibodies	Species	Manufacturer	Catalog #	IF dilution	Western blot dilution
Anti-Occludin	Rabbit	Abcam	ab216327	1:200	1:1000
Anti-BCRP	Rat	Enzo	ALX-801-036-C100	1:30	
Anti-CD31	Rabbit	Abcam	ab28364	1:30	
Anti-CD31	Rat	Santa Cruz	sc-18916	1:50	
Anti-Claudin5	Mouse	Invitrogen	4C3C2	1:125	1:500
Anti-PGP	Mouse	Santa Cruz	sc-13131	1:20	
Anti-ZO1/TJ1	Rabbit	Invitrogen	61-7300	1:35	1:666

### Animal studies

All animal experiments were conducted in accordance with legal and ethical regulations and approved by the responsible council (Germany, Regierungspräsidium Karlsruhe: G-164/17, G-187/18, G-227/19, G-228/19, G-255/19, G-76/20, G-58/22 and G-91/20). The study was performed according to GV-SOLAS and ARRIVE guidelines. Mice were obtained from in-house breeding of the DKFZ (Heidelberg, Germany), housed in IVC caging in the Center for Preclinical Research of the DKFZ (Heidelberg, Germany) and monitored daily for the presence of tumor-related symptoms. Cohort sizes were chosen to minimize the number of animals required to get statistically significant results.

### Patient-derived xenograft mouse models

Patient-derived tumor cells were orthotopically injected in 6–8 week old immunocompromised mice (NOD-SCID gamma mice (NSG)). Before injections, experimental animals received a painkiller (Metamizol, s.c., 200 mg/kg or 5 mg/kg s.c. Carprofen). Mice were sedated with 1.5–2.5 Vol% isoflurane and eye ointment avoided dehydration. After a negative reflex test, mice were placed on a heating mat with stereotactic fixation. The head was disinfected, a 5 mm incision was performed, and a whole was drilled in the scull at the right position using coordinates from bregma (front suture intersection) and lambda (ZFTA cells in cortex and PFA cells in cerebellum). Tumor cells were intracranially injected with a hamilton needle. After a short waiting time and prior to retracting the needle, the wound was closed with tissue adhesive histoacryl (Braun, Germany). After surgery mice received painkiller via drinking water for three days.

### In utero electroporation (IUE) mouse models

DNA fusion gene constructs were transfected into CD1 mice at E13.5 as described in Zheng et al.^68^. After birth, mice developed tumors within days to weeks depending on the exact model. Tumor growth was measured via bioluminescence imaging or MRI techniques.

### RNA-sequencing data processing and downstream analysis

A combined genome reference was constructed with human reference genome GRCh38 and mouse reference GRCm38 using STAR (version 2.7.10b). Raw sequencing reads were aligned to the combined reference with STAR. Count matrices were generated subsequently with featureCounts (version 2.0.6) and a combined annotation file. Counts were then separated back into human counts and mouse counts by gene annotation. This dataset was combined with an independent human patient cohort from the INFORM database^30^ and an IUE mouse model cohort as summarized in Table S3. The patient part of the data (n = 106) evaluated in this study was produced and kindly provided by the INFORM program.

Species specific counts were further processed using DESeq2 (version 1.46.0) in R (version 4.4.3). Size factors were estimated to normalize for sequencing depth and differences in library size across samples. Normalized counts were further transformed using variance stabilization transformation (vst) to assure constant variance along the range of mean values. Visualization of normalized and raw counts was performed using R packages ggplot2 (version 3.5.2) and pheatmap (version 1.0.13). After normalization, datasets were merged and subsetted by the respective marker genes. t-distributed Stochastic Neighbor Embedding (tSNE) was subsequently applied for dimensionality reduction via Rtsne package (version 0.17).

### Bioluminescence imaging

Bioluminescence measurements were performed to monitor tumor size developments in mouse models after tumor cell labelling with luciferase. Mice were sedated with 1.5–2 Vol% isoflurane, luciferin solution (15 mg/ml) was injected intraperitoneally (i.p.) accordingly to the body weight and photons per seconds were measured.

### Magnet resonance imaging (MRI)

MRI was carried out at the small animal imaging core facility at the DKFZ using a BioSpec 3 Tesla (Bruker, Germany) with ParaVision software 360 V1.1. For the imaging, mice were anesthetized with 3.5% sevoflurane in air. For lesion detection T2 weighted imaging were performed using a T2_TurboRARE sequence: TE = 48 ms, TR = 3350 ms, FOV 20 × 20 mm, slice thickness 1 mm, averages = 3, Scan Time 3m21s, echo spacing 12 ms, rare factor 8, slices 20, image size 192 × 192. Tumor volume was measured using a T1-FLASH sequence with contrast agent, 80–100 µl ProHance (Bracco Imaging, Germany) i.p.): TE = 3ms, TR = 500ms, FOV 20 × 20 mm, slice thickness 1 mm, slices 20, Flip angle 70, averages = 3, resolution = 0,104 mm. Tumor volume was determined by manual segmentation using Bruker ParaVision software 6.0.1. The regions of interest (ROI) were visualized and manually labelled using a the RadiAnt DICOM Viewer Software.

### Ultramicroscopy

In order to label endothelial cells, Lectin-TexasRed (12 mg/kg body weight, Vector Laboratories; USA) was injected intravenously into mice prior to sedation. Cardiac perfusion was performed with sodium fluorescein (Sigma, Germany). Next, mice were perfused with 4% PFA and brains were fixed in 4% PFA for 24 h at RT, afterwards the brain was transferred in ascending butanol solutions (30%, 50%, 70%, 80%, 96%, 100%, 100%), each for 24 h at RT^69^. After refractive index matching and lipid removal in benzyl alcohol benzyl benzoate (BABB) solution, tissue was transferred in ethyl cinnamate for imaging in lightsheet 7 (Zeiss, Germany) with 20x objective.

### Mouse treatment study

EPN PDX mouse models with a tumor bioluminescence signal of at least 3 × 10^6^ photons/seconds were treated daily for three consecutive days with idasanutlin (Hycultec, Germany, 150 mg/kg, i.p.) and temsirolimus (Alsachim, France, 150 mg/kg, i.p.). 4 h after the last treatment, mice were euthanized by increasing CO2 concentrations. Etoposide (Biozol, Germany, 20 mg/kg), i.p. was given daily for three consecutive days and on the last day combined with carboplatin (Biozol, Germany, 50 mg/kg, i.v.). Mice were euthanized 0.5–1 h afterwards. Post mortem, blood was taken by cardiac puncture and brain tissue was collected for pharmacokinetic measurements after PBS perfusion separated into cortex, cerebellum and tumor.

### Mouse brain tissue homogenization and drug extraction

1 mg of brain tissue was dissolved in 10 µL of water/acetonitrile (H_2_O/ACN, 95/5) solution supplemented with 30 glass beads (0.75–1 mm; Carl Roth GmbH, Germany). Tissue samples were homogenized using the Bead Ruptor 4 homogenizer (Omni International Inc, USA) for at least 30 s and stored at -20 °C.

### Development of UPLC-MS/MS methods for drugs quantification

Stock solution of idasanutlin (Hycultec, Germany) was prepared in 1:1 H2O/ACN, and calibration/quality control solutions were prepared in 1:1 H2O/ACN + 0.1% FA. The stock solution of internal standard idasanutlin-d3-1 (Hycultec, Germany) was dissolved in ethanol and working solution was dissolved in 1:1 H2O/ACN. After plasma protein precipitation with acetonitrile, the samples were subjected to chromatographic separation on an ACQUITY UPLC BEH C18 column (Waters, USA) with an acetonitrile gradient. For UPLC-MS/MS quantification, a triple-stage quadrupole mass spectrometer (Waters Xevo TQ-S with Z-spray electrospray ionization (ESI) source) with an Acquity Classic UPLC^®^ (Waters, Milford, MA, USA) was used. Idasanutlin calibration range was determined between 0.3 and 1000 ng/mL and 3–10,000 ng/g for mouse plasma and brain homogenate, respectively.

Stock solutions, calibration solutions and quality control samples of temsirolimus (Alsachim, France) and its internal standard temsirolimus-13C3,2H7 (Alsachim, France) were prepared in 1:1 H2O/ACN. After whole blood protein precipitation with acetonitrile, the samples were subjected to chromatographic separation on an ACQUITY UPLC BEH C18 column (Waters, USA) with an acetonitrile gradient. For UPLC-MS/MS quantification the same device as described for idasanutlin was used. Temsirolimus calibration range was determined between 10 and 10,000 ng/mL and 100–100,000 ng/g in mouse whole blood and brain homogenate, respectively.

Stock solutions of etoposide (Med Chem Express, USA) and its internal standard etoposide-d3 (Santa Cruz Biotechnology, USA) were diluted in 1:1 H2O/ACN. Calibration/quality control solutions, and working solution of the internal standard were prepared in 95/5 H_2_O/ACN + 0.1% FA. After plasma protein precipitation with acetonitrile, the samples were subjected to chromatographic separation on an ACQUITY UPLC BEH C18 column (Waters, USA) with an acetonitrile gradient in the presence of 5 mM ammonium acetate. For UPLC-MS/MS quantification, a triple-stage quadrupole mass spectrometer (Waters Xevo TQ-XS with Z-spray electrospray ionization (ESI) source) with an Acquity Classic UPLC^®^ (Waters, Milford, MA, USA) was used. Etoposide calibration range was determined between 3 − 1,000ng/mL and 30 − 10,000 ng/g in plasma and brain homogenate, respectively.

### Western blot

Mouse tissues were homogenized in cold PBS by using a tissue homogenizer for 30 s. After centrifugation of 10 min at 4 °C 300 g, the pellet was dissolved in 150 µl RIPA buffer containing the protease inhibitor cocktail cOmplete (Merck, Germany) and incubated for 1 h on ice. After lysis, cell homogenate was centrifuged 10 min at 4 °C 16,200 g. Proteins in supernatant were separated and blotted as described in Okonechnikov et al.^70^. Membranes were blocked for 1 h with 5% BSA except for TJP1 antibody, for which 5% milk in Tris buffered saline with Tween20 (TBS-T). They were then incubated overnight at 4 °C with the primary antibody (Table 2), followed by washing and 1 h incubation in secondary horseradish peroxidase-conjugated antibodies (cell signaling, dilution 1:2500).

### Sodium fluorescein and endothelial cell quantification

For sodium fluorescein intensity quantification, imagej was used to substract lectin staining and remaining intensity in all z-stack images was quantified.

Endothelial cell quantification from human/mouse immunofluorescence images were performed with cellprofiler 4.2.6 using identify primary, secondary, tertiary objects followed by measureImageAreaOccupied.

## Supplementary Information

Below is the link to the electronic supplementary material.

Supplementary Material 1

Supplementary Material 2

## Data Availability

Human RNA array data are publicly available (GEO: GSE64415 and GEO: GSE50161, GSE50385, GSE21687, GSE3526). All other data are available from the corresponding author upon request.

## Code availability

Code for single cell analysis is available here: https://github.com/afederico-sci/scRNAseq_BBB_analysis.

## References

[1] Gawdi, R., Shumway, K. R. & Emmady, P. D. in *StatPearls* (StatPearls Publishing Copyright © 2022, StatPearls Publishing LLC., (2022).

[2] Abbott, N. J., Rönnbäck, L. & Hansson, E. Astrocyte-endothelial interactions at the blood-brain barrier. *Nat. Rev. Neurosci.* **7**, 41–53. 10.1038/nrn1824 (2006). 16371949 10.1038/nrn1824

[3] Bagchi, S. et al. In-vitro blood-brain barrier models for drug screening and permeation studies: an overview. *Drug Des. Devel Ther.* **13**, 3591–3605. 10.2147/dddt.s218708 (2019). 31695329 10.2147/DDDT.S218708 PMC6805046

[4] Sweeney, M. D., Zhao, Z., Montagne, A., Nelson, A. R. & Zlokovic, B. V. Blood-brain barrier: from physiology to disease and back. *Physiol. Rev.* **99**, 21–78. 10.1152/physrev.00050.2017 (2019). 30280653 10.1152/physrev.00050.2017 PMC6335099

[5] Silwedel, C. & Förster, C. Differential susceptibility of cerebral and cerebellar murine brain microvascular endothelial cells to loss of barrier properties in response to inflammatory stimuli. *J. Neuroimmunol.* **179**, 37–45. 10.1016/j.jneuroim.2006.06.019 (2006). https://doi.org: 16884785 10.1016/j.jneuroim.2006.06.019

[6] Cummins, M. J., Cresswell, E. T., Bevege, R. J. & Smith, D. W. Aging disrupts blood–brain and blood-spinal cord barrier homeostasis, but does not increase paracellular permeability. *GeroScience* **47**, 263–285. 10.1007/s11357-024-01404-9 (2025). 39476323 10.1007/s11357-024-01404-9 PMC11872845

[7] Ostrom, Q. T. et al. CBTRUS statistical report: primary brain and other central nervous system tumors diagnosed in the United States in 2016–2020. *Neuro-Oncol.* **25**, iv1–iv99. 10.1093/neuonc/noad149 (2023). 10.1093/neuonc/noad149 PMC10550277 37793125

[8] Gribkoff, V. K. & Kaczmarek, L. K. The need for new approaches in CNS drug discovery: Why drugs have failed, and what can be done to improve outcomes. *Neuropharmacology* **120**, 11–19. 10.1016/j.neuropharm.2016.03.021 (2017). 26979921 10.1016/j.neuropharm.2016.03.021 PMC5820030

[9] Pardridge, W. M. & Boado, R. J. Reengineering biopharmaceuticals for targeted delivery across the blood-brain barrier. *Methods Enzymol.* **503**, 269–292. 10.1016/b978-0-12-396962-0.00011-2 (2012). 22230573 10.1016/B978-0-12-396962-0.00011-2

[10] Muldoon, L. L. et al. Chemotherapy delivery issues in central nervous system malignancy: a reality check. *J. Clin. Oncol.* **25**, 2295–2305. 10.1200/jco.2006.09.9861 (2007). 17538176 10.1200/JCO.2006.09.9861

[11] Orthmann, A., Fichtner, I. & Zeisig, R. Improving the transport of chemotherapeutic drugs across the blood–brain barrier. *Expert Rev. Clin. Pharmacol.* **4**, 477–490. 10.1586/ecp.11.26 (2011). 22114857 10.1586/ecp.11.26

[12] Lee, G., Dallas, S., Hong, M. & Bendayan, R. Drug transporters in the central nervous system: brain barriers and brain parenchyma considerations. *Pharmacol. Rev.* **53**, 569–596 (2001). 11734619

[13] Pajtler, K. W. et al. Molecular classification of ependymal tumors across all CNS compartments, histopathological grades, and age groups. *Cancer Cell.* **27**, 728–743. 10.1016/j.ccell.2015.04.002 (2015). 25965575 10.1016/j.ccell.2015.04.002 PMC4712639

[14] Ghasemi, D. R. et al. MYCN amplification drives an aggressive form of spinal ependymoma. *Acta Neuropathol.* **138**, 1075–1089. 10.1007/s00401-019-02056-2 (2019). 31414211 10.1007/s00401-019-02056-2 PMC6851394

[15] Phoenix, T. N. et al. Medulloblastoma genotype dictates blood brain barrier phenotype. *Cancer Cell.* **29**, 508–522. 10.1016/j.ccell.2016.03.002 (2016). 27050100 10.1016/j.ccell.2016.03.002 PMC4829447

[16] Millard, N. E. & De Braganca, K. C. Medulloblastoma. *J. Child. Neurol.* **31**, 1341–1353 10.1177/0883073815600866 (2016). 26336203 10.1177/0883073815600866 PMC4995146

[17] Chapman, R. J. et al. Optimizing biomarkers for accurate ependymoma diagnosis, prognostication, and stratification within International Clinical Trials: A BIOMECA study. *Neuro Oncol.* **25**, 1871–1882. 10.1093/neuonc/noad055 (2023). 36916248 10.1093/neuonc/noad055 PMC10547510

[18] Greene, C. & Campbell, M. Tight junction modulation of the blood brain barrier: CNS delivery of small molecules. *Tissue Barriers*. **4**, e1138017. 10.1080/21688370.2015.1138017 (2016). 27141420 10.1080/21688370.2015.1138017 PMC4836485

[19] Stamatovic, S. M., Johnson, A. M., Keep, R. F. & Andjelkovic, A. V. Junctional proteins of the blood-brain barrier: new insights into function and dysfunction. *Tissue Barriers*. **4**, e1154641. 10.1080/21688370.2016.1154641 (2016). 27141427 10.1080/21688370.2016.1154641 PMC4836471

[20] Bhowmik, A., Khan, R. & Ghosh, M. K. Blood brain barrier: a challenge for effectual therapy of brain tumors. *Biomed. Res. Int.* **2015**, 320941. 10.1155/2015/320941 (2015). 10.1155/2015/320941 PMC4383356 25866775

[21] Tietz, S. & Engelhardt, B. Brain barriers: crosstalk between complex tight junctions and adherens junctions. *J. Cell. Biol.* **209**, 493–506. 10.1083/jcb.201412147 (2015). 26008742 10.1083/jcb.201412147 PMC4442813

[22] Berndt, P. et al. Tight junction proteins at the blood-brain barrier: far more than claudin-5. *Cell. Mol. Life Sci.* **76**, 1987–2002. 10.1007/s00018-019-03030-7 (2019). 30734065 10.1007/s00018-019-03030-7 PMC11105330

[23] Schossleitner, K. et al. Evidence that cingulin regulates endothelial barrier function in vitro and in vivo. *Arterioscler. Thromb. Vasc. Biol.* **36**, 647–654. 10.1161/atvbaha.115.307032 (2016). 26821949 10.1161/ATVBAHA.115.307032

[24] Roth, R. B. et al. Gene expression analyses reveal molecular relationships among 20 regions of the human CNS. *Neurogenetics* **7**, 67–80. 10.1007/s10048-006-0032-6 (2006). 16572319 10.1007/s10048-006-0032-6

[25] Okonechnikov, K. et al. Mapping pediatric brain tumors to their origins in the developing cerebellum. *Neuro Oncol.* **25**, 1895–1909. 10.1093/neuonc/noad124 (2023). 37534924 10.1093/neuonc/noad124 PMC10547518

[26] Cardoso-Moreira, M. et al. Gene expression across mammalian organ development. *Nature* **571**, 505–509. 10.1038/s41586-019-1338-5 (2019). 31243369 10.1038/s41586-019-1338-5 PMC6658352

[27] Gojo, J. et al. Single-cell RNA-Seq reveals cellular hierarchies and impaired developmental trajectories in pediatric ependymoma. *Cancer Cell* **38**, 44–59.e49. 10.1016/j.ccell.2020.06.004 (2020). 10.1016/j.ccell.2020.06.004 PMC7479515 32663469

[28] Aubin, R. G. et al. Pro-inflammatory cytokines mediate the epithelial-to-mesenchymal-like transition of pediatric posterior fossa ependymoma. *Nat. Commun.* **13**, 3936. 10.1038/s41467-022-31683-9 (2022). 35803925 10.1038/s41467-022-31683-9 PMC9270322

[29] Xie, Y. et al. Key molecular alterations in endothelial cells in human glioblastoma uncovered through single-cell RNA sequencing. *JCI Insight*. e1138017. 10.1172/jci.insight.150861 (2021). 10.1172/jci.insight.150861 PMC8410070 34228647

[30] van Tilburg, C. M. et al. The pediatric precision oncology INFORM registry: clinical outcome and benefit for patients with very high-evidence targets. *Cancer Discov.* **11**, 2764–2779. 10.1158/2159-8290.Cd-21-0094 (2021). 34373263 10.1158/2159-8290.CD-21-0094 PMC9414287

[31] Rudà, R., Bruno, F., Pellerino, A. & Soffietti, R. Ependymoma: evaluation and management updates. *Curr. Oncol. Rep.* **24**, 985–993. 10.1007/s11912-022-01260-w (2022). 35384591 10.1007/s11912-022-01260-w PMC9249684

[32] Deeken, J. F. & Löscher, W. The blood-brain barrier and cancer: transporters, treatment, and trojan horses. *Clin. Cancer Res.* **13**, 1663–1674. 10.1158/1078-0432.Ccr-06-2854 (2007). 17363519 10.1158/1078-0432.CCR-06-2854

[33] Pajouhesh, H. & Lenz, G. R. Medicinal chemical properties of successful central nervous system drugs. *NeuroRx* **2**, 541–553. 10.1602/neurorx.2.4.541 (2005). 16489364 10.1602/neurorx.2.4.541 PMC1201314

[34] Vellonen, K. S. et al. Disease-induced alterations in brain drug transporters in animal models of Alzheimer’s disease. *Pharm. Res.* **34**, 2652–2662. 10.1007/s11095-017-2263-7 (2017). 28952054 10.1007/s11095-017-2263-7

[35] Sharma, K. et al. Cell type– and brain region–resolved mouse brain proteome. *Nat. Neurosci.* **18**, 1819–1831. 10.1038/nn.4160 (2015). 26523646 10.1038/nn.4160 PMC7116867

[36] Schumacher, U. & Mollgård, K. The multidrug-resistance P-glycoprotein (Pgp, MDR1) is an early marker of blood-brain barrier development in the microvessels of the developing human brain. *Histochem. Cell. Biol.* **108**, 179–182. 10.1007/s004180050159 (1997). 9272437 10.1007/s004180050159

[37] Ek, C. J. et al. Efflux mechanisms at the developing brain barriers: ABC-transporters in the fetal and postnatal rat. *Toxicol. Lett.* **197**, 51–59. 10.1016/j.toxlet.2010.04.025 (2010). 20466047 10.1016/j.toxlet.2010.04.025

[38] Bors, L. et al. Age-dependent changes at the blood-brain barrier. A comparative structural and functional study in young adult and middle aged rats. *Brain Res. Bull.* **139**, 269–277. 10.1016/j.brainresbull.2018.03.001 (2018). 29522862 10.1016/j.brainresbull.2018.03.001

[39] Ghersi-Egea, J. F., Saudrais, E. & Strazielle, N. Barriers to drug distribution into the perinatal and postnatal brain. *Pharm. Res.* **35**, 84. 10.1007/s11095-018-2375-8 (2018). 29516182 10.1007/s11095-018-2375-8

[40] Martin, T. A., Mason, M. D. & Jiang, W. G. Tight junctions in cancer metastasis. *Front. Biosci.* **16**, 898–936 (2013). 10.2741/3726 21196209

[41] Osanai, M. et al. Occludin expression inhibits tumorigenicity and metastasis. *FASEB J.* **20** A223-A223 (2006).

[42] Rivero-Hinojosa, S. et al. Proteomic analysis of Medulloblastoma reveals functional biology with translational potential. *Acta Neuropathol. Commun.* **6**, 48. 10.1186/s40478-018-0548-7 (2018). 29880060 10.1186/s40478-018-0548-7 PMC5992829

[43] Upadhya, S. R. & Ryan, C. J. Experimental reproducibility limits the correlation between mRNA and protein abundances in tumor proteomic profiles. *Cell. Rep. Methods*. **2**, 100288. 10.1016/j.crmeth.2022.100288 (2022). 36160043 10.1016/j.crmeth.2022.100288 PMC9499981

[44] Goodall, E. F. et al. Age-associated changes in the blood-brain barrier: comparative studies in human and mouse. *Neuropathol. Appl. Neurobiol.* **44**, 328–340. 10.1111/nan.12408 (2018). 28453876 10.1111/nan.12408 PMC5900918

[45] Genovesi, L. A. et al. Patient-derived orthotopic xenograft models of medulloblastoma lack a functional blood-brain barrier. *Neuro Oncol.* **23**, 732–742. 10.1093/neuonc/noaa266 (2021). 33258962 10.1093/neuonc/noaa266 PMC8099473

[46] Zhao, Z., Nelson, A. R., Betsholtz, C. & Zlokovic, B. V. Establishment and dysfunction of the blood-brain barrier. *Cell* **163**, 1064–1078. 10.1016/j.cell.2015.10.067 (2015). 26590417 10.1016/j.cell.2015.10.067 PMC4655822

[47] Karnati, H. K. et al. Down regulated expression of Claudin-1 and Claudin-5 and up regulation of β-catenin: association with human glioma progression. *CNS Neurol. Disord. Drug Targets*. **13**, 1413–1426. 10.2174/1871527313666141023121550 (2014). 25345514 10.2174/1871527313666141023121550 PMC6138250

[48] Nitta, T. et al. Size-selective loosening of the blood-brain barrier in claudin-5-deficient mice. *J. Cell. Biol.* **161**, 653–660. 10.1083/jcb.200302070 (2003). 12743111 10.1083/jcb.200302070 PMC2172943

[49] Liu, F. et al. Dihydroartemisinin protects blood-brain barrier permeability during sepsis by inhibiting the transcription factor SNAI1. *Clin. Exp. Pharmacol. Physiol.* **49**, 979–987. 10.1111/1440-1681.13683 (2022). 35651290 10.1111/1440-1681.13683 PMC9543489

[50] Malgulwar, P. B. et al. Transcriptional co-expression regulatory network analysis for Snail and Slug identifies IL1R1, an inflammatory cytokine receptor, to be preferentially expressed in ST-EPN-RELA and PF-EPN-A molecular subgroups of intracranial ependymomas. *Oncotarget* **9**, 35480–35492. 10.18632/oncotarget.26211 (2018). 30464804 10.18632/oncotarget.26211 PMC6231457

[51] Zhang, M. et al. Radiomics can distinguish pediatric supratentorial embryonal tumors, high-grade gliomas, and ependymomas. *AJNR Am. J. Neuroradiol.* **43**, 603–610. 10.3174/ajnr.A7481 (2022). 35361575 10.3174/ajnr.A7481 PMC8993189

[52] Kim, K. J., Park, M., Joo, B., Ahn, S. J. & Suh, S. H. Dynamic contrast-enhanced MRI and its applications in various central nervous system diseases. *Investig Magn. Reson. Imaging*. **26**, 256–264 (2022).

[53] Gupta, P. K. et al. Role of dynamic contrast-enhanced perfusion magnetic resonance imaging in grading of pediatric brain tumors on 3T. *Pediatr. NeuroSurg.* **52**, 298–305. 10.1159/000479283 (2017). 28848203 10.1159/000479283

[54] Hill, C. R. et al. Characterisation of the roles of ABCB1, ABCC1, ABCC2 and ABCG2 in the transport and pharmacokinetics of actinomycin D in vitro and in vivo. *Biochem. Pharmacol.* **85**, 29–37. 10.1016/j.bcp.2012.10.004 (2013). 23063411 10.1016/j.bcp.2012.10.004 PMC3545186

[55] Mai, W. X. et al. Cytoplasmic p53 couples oncogene-driven glucose metabolism to apoptosis and is a therapeutic target in glioblastoma. *Nat. Med.* **23**, 1342–1351. 10.1038/nm.4418 (2017). 29035366 10.1038/nm.4418 PMC5683421

[56] Lagas, J. S. et al. P-glycoprotein (P-gp/Abcb1), Abcc2, and Abcc3 determine the pharmacokinetics of etoposide. *Clin. Cancer Res.* **16**, 130–140. 10.1158/1078-0432.Ccr-09-1321 (2010). 20028753 10.1158/1078-0432.CCR-09-1321

[57] Reis, F. R. et al. Survivin and P-glycoprotein are associated and highly expressed in late phase chronic myeloid leukemia. *Oncol. Rep.* **26**, 471–478. 10.3892/or.2011.1296 (2011). 21567097 10.3892/or.2011.1296

[58] Mandery, K., Glaeser, H. & Fromm, M. F. Interaction of innovative small molecule drugs used for cancer therapy with drug transporters. *Br. J. Pharmacol.* **165**, 345–362. 10.1111/j.1476-5381.2011.01618.x (2012). 21827448 10.1111/j.1476-5381.2011.01618.x PMC3268189

[59] Shamsi, A. et al. Investigating the interaction of anticancer drug temsirolimus with human transferrin: molecular docking and spectroscopic approach. *J. Mol. Recogn.* **31**, e2728. 10.1002/jmr.2728 (2018). 10.1002/jmr.2728 29770561

[60] Pajtler, K. W. et al. YAP1 subgroup supratentorial ependymoma requires TEAD and nuclear factor I-mediated transcriptional programmes for tumorigenesis. *Nat. Commun.* **10**, 3914. 10.1038/s41467-019-11884-5 (2019). 31477715 10.1038/s41467-019-11884-5 PMC6718408

[61] Brabetz, S. et al. A biobank of patient-derived pediatric brain tumor models. *Nat. Med.* **24**, 1752–1761. 10.1038/s41591-018-0207-3 (2018). 30349086 10.1038/s41591-018-0207-3

[62] Drokhlyansky, E. et al. The Human and Mouse Enteric Nervous System at Single-Cell Resolution. *Cell* **182** (6), 1606–1622. 10.1016/j.cell.2020.08.003(2020). 32888429 10.1016/j.cell.2020.08.003 PMC8358727

[63] Slyper, M. et al. A single-cell and single-nucleus RNA-Seq toolbox for fresh and frozen human tumors. *Nat. Med.* **26**, 792–802. 10.1038/s41591-020-0844-1 (2020). 32405060 10.1038/s41591-020-0844-1 PMC7220853

[64] Lago, C. et al. Patient- and xenograft-derived organoids recapitulate pediatric brain tumor features and patient treatments. *EMBO Mol. Med.* **15**, e18199. 10.15252/emmm.202318199 (2023). 38037472 10.15252/emmm.202318199 PMC10701620

[65] Korsunsky, I. et al. Fast, sensitive and accurate integration of single-cell data with Harmony. *Nat. Methods*. **16**, 1289–1296. 10.1038/s41592-019-0619-0 (2019). 31740819 10.1038/s41592-019-0619-0 PMC6884693

[66] Blanco-Carmona, E. Generating publication ready visualizations for single cell transcriptomics using SCpubr. *bioRxiv* **2022.2002.2028.482303**10.1101/2022.02.28.482303 (2022).

[67] Scheuermann, S. et al. Unveiling spatial complexity in solid tumor immune microenvironments through multiplexed imaging. *Front. Immunol.* **15**, 1383932. 10.3389/fimmu.2024.1383932 (2024). 38566984 10.3389/fimmu.2024.1383932 PMC10985204

[68] Zheng, T. et al. Cross-species genomics reveals oncogenic dependencies in ZFTA/C11orf95 fusion-positive supratentorial ependymomas. *Cancer Discov*. **11**, 2230–2247. 10.1158/2159-8290.cd-20-0963 (2021). 33879448 10.1158/2159-8290.CD-20-0963

[69] Schwarz, M. K. et al. Fluorescent-protein stabilization and high-resolution imaging of cleared, intact mouse brains. *PLOS One*. **10**, e0124650. 10.1371/journal.pone.0124650 (2015). 25993380 10.1371/journal.pone.0124650 PMC4439039

[70] Okonechnikov, K. et al. 3D genome mapping identifies subgroup-specific chromosome conformations and tumor-dependency genes in ependymoma. *Nat. Commun.* **14**, 2300. 10.1038/s41467-023-38044-0 (2023). 37085539 10.1038/s41467-023-38044-0 PMC10121654

[71] Worst B.C. et al. Next-generation personalised medicine for high-risk paediatric cancer patients – The INFORM pilot study European Journal of Cancer 6591–101. 10.1016/j.ejca.2016.06.009 (2016) 27479119 10.1016/j.ejca.2016.06.009

[72] Peterziel, H. et al. Drug sensitivity profiling of 3D tumor tissue cultures in the pediatric precision oncology program INFORM. *NPJ Precis Oncol.* **6** (1), 94 (2022). 36575299 10.1038/s41698-022-00335-y PMC9794727

[73] Heipertz, A. E. et al. Outcome of Children and Adolescents With Relapsed/Refractory/Progressive Malignancies Treated With Molecularly Informed Targeted Drugs in the Pediatric Precision Oncology Registry INFORM. *JCO Precis Oncol.* **7**, e2300015 (2023). 37364231 10.1200/PO.23.00015

