# Supplementary material for: Ependymoma group-specific blood-brain barrier differences uncovered by a multi-omics approach
Source: Sci Rep. 2026 Apr 10;16:12061. doi: 10.1038/s41598-026-47499-2 (PMC13070042; doi:10.1038/s41598-026-47499-2)
Supplement: Supplementary file 2 — Supplementary Material 2 [file 41598_2026_47499_MOESM2_ESM.docx]

**
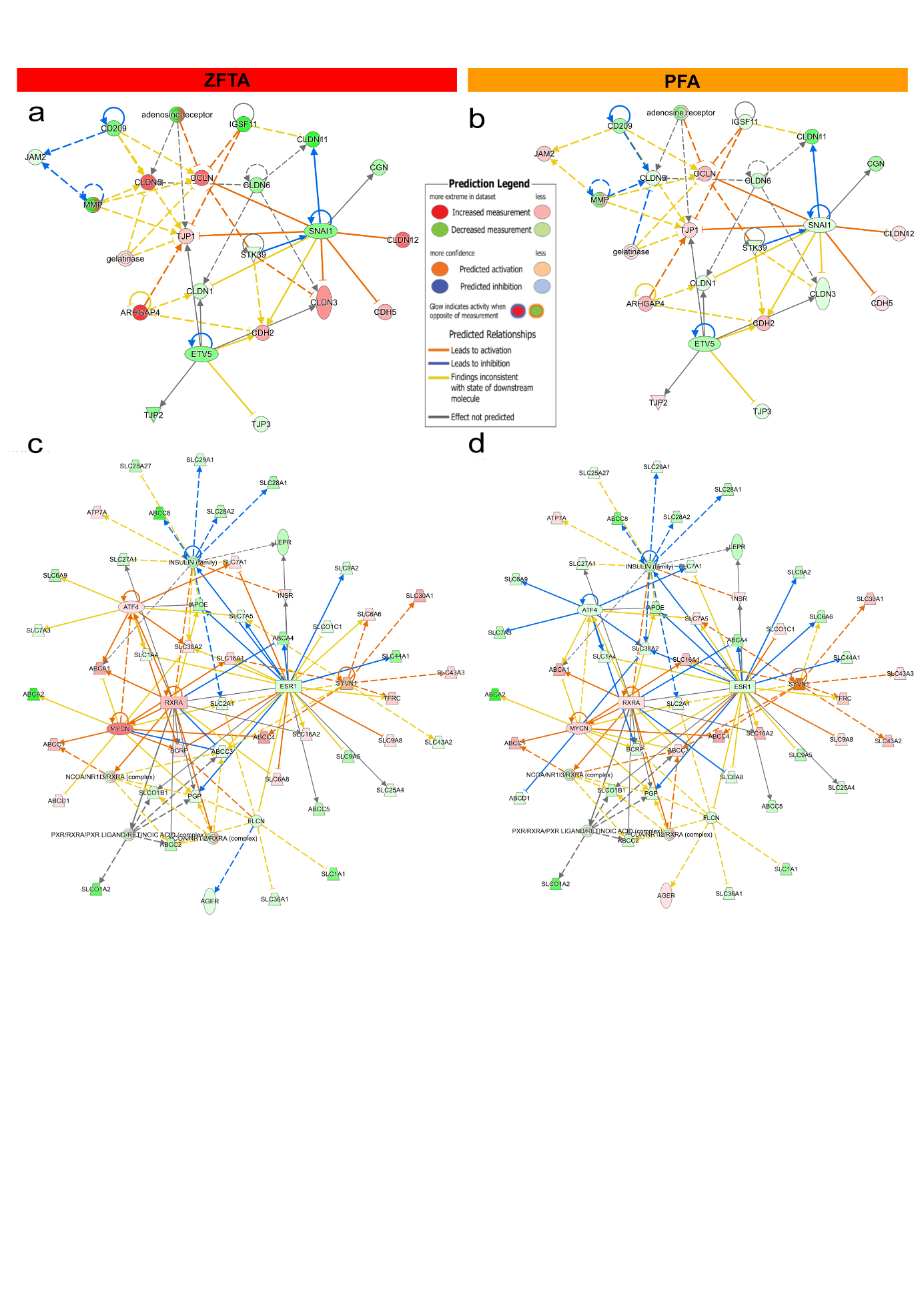
**

Figure S1: **Interactive pathway analysis (IPA) highlighting altered expression of the top 10 upstream regulators of BBB genes a** Tight junction regulators in ZFTA or **b** PFA and **c** transporter regulators in ZFTA or **d** PFA.

**
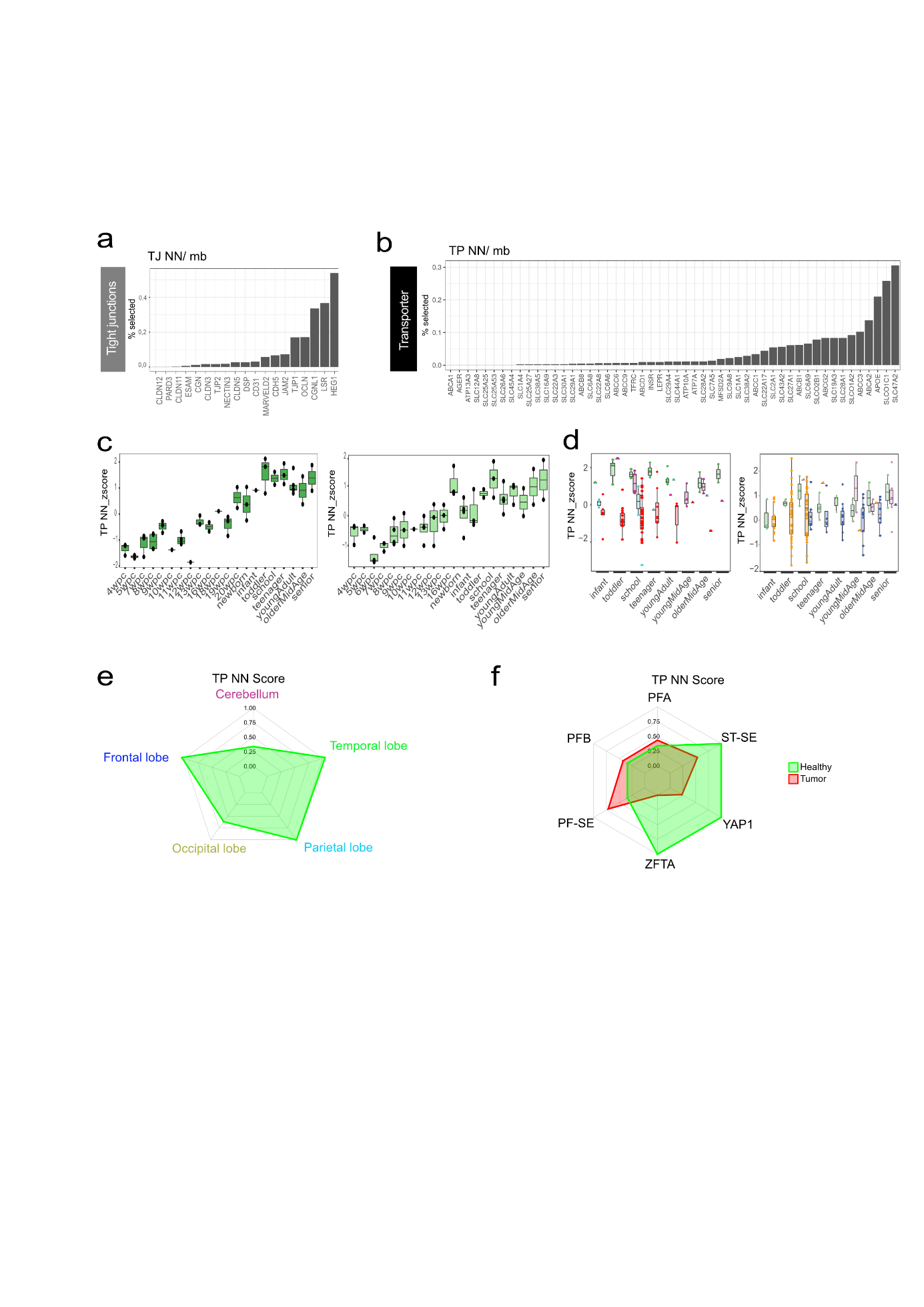
**

Figure S2: **Integration of tight junction and transporter gene sets into neural network (NN) analysis to establish TJ NN and a TP NN scores.** **a** Tight junction genes with highest weights contributing to the TJ NN score. **b** Transporter and receptor genes with the highest weights contributing to the TP NN score. **c** TP NN score in healthy cortex and cerebellum at different development stages and **d** in different EPN tumor tissues located in cortex or cerebellum. **e** TP NN scores for healthy brain regions and **f** EPN tumor tissue.


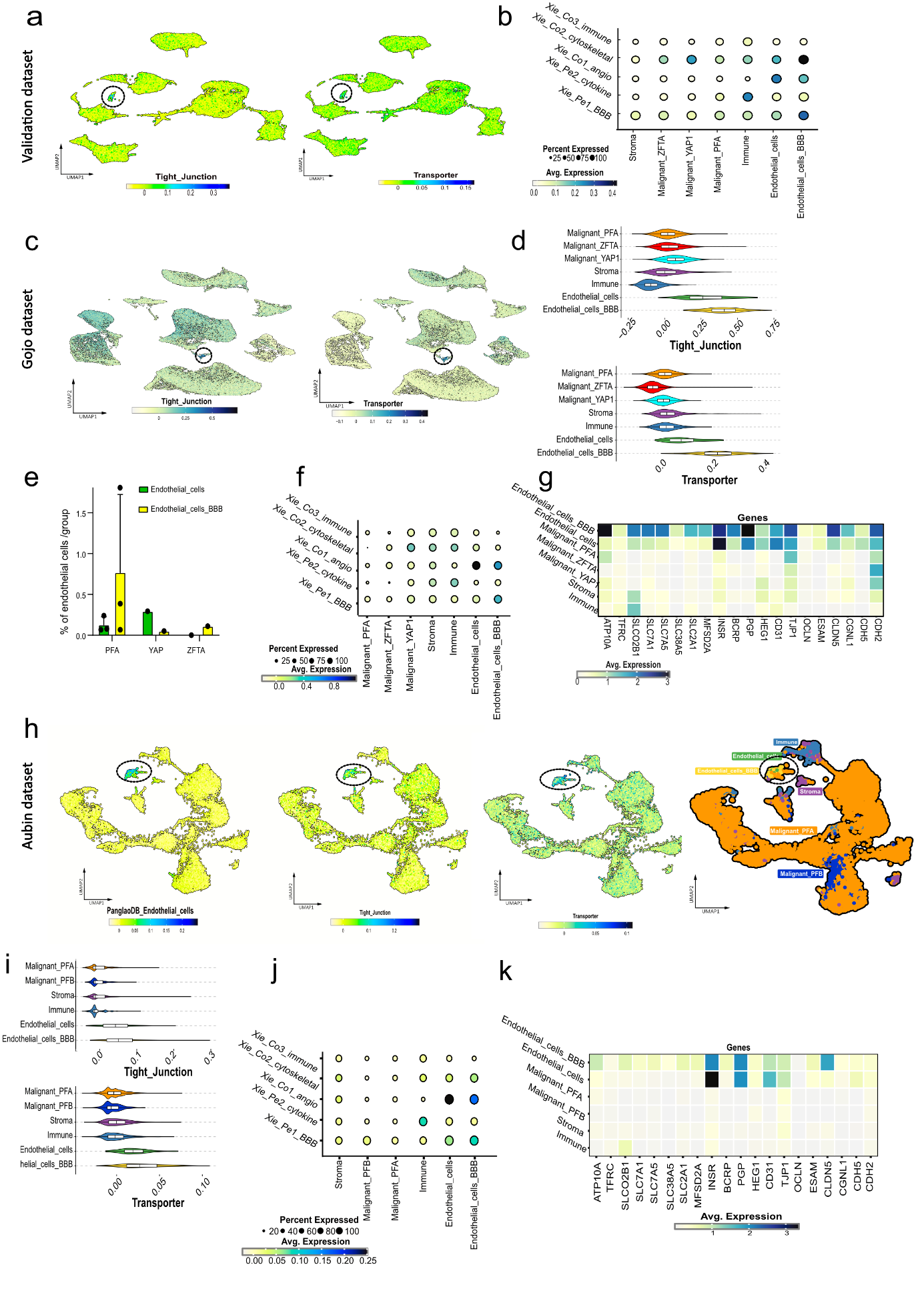
Figure S3: **Single-cell re-analyses of the EPN dataset from Gojo and Aubin differentiating malignant and endothelial cell clusters.** **a** UMAP showing the score intensity of tight junction (right) and transporter signatures (left) in the validation dataset. **b** Dot plot of GBM-informed endothelial gene lists with average expression across EPN clusters in the validation dataset. **c** UMAP showing tight junction (right) and transporter (left) expression in the Gojo dataset. **d** Gene enrichment violin plot of tight junction and transporter gene lists across all clusters. **e** Bar plot of the two endothelial clusters from the Gojo dataset. **f** Dot plot of GBM informed endothelial-gene lists with average expression across EPN clusters from the Gojo dataset. **g** Heatmap of the most enriched tight junction and transporter genes from the Gojo dataset. **h** UMAP showing expression intensity of Pangloa endothelial cell, tight junction, and transporter signatures with annotated cell types from the Aubin dataset. **i** Gene enrichment violin plot of tight junction and transporter signatures across EPN clusters from Aubin dataset. **j** Dot plot of GBM-informed endothelial cell gene lists with average expression across EPN clusters from the Aubin dataset. **k** Heatmap of the most enriched tight junction and transporter genes from the Aubin dataset.


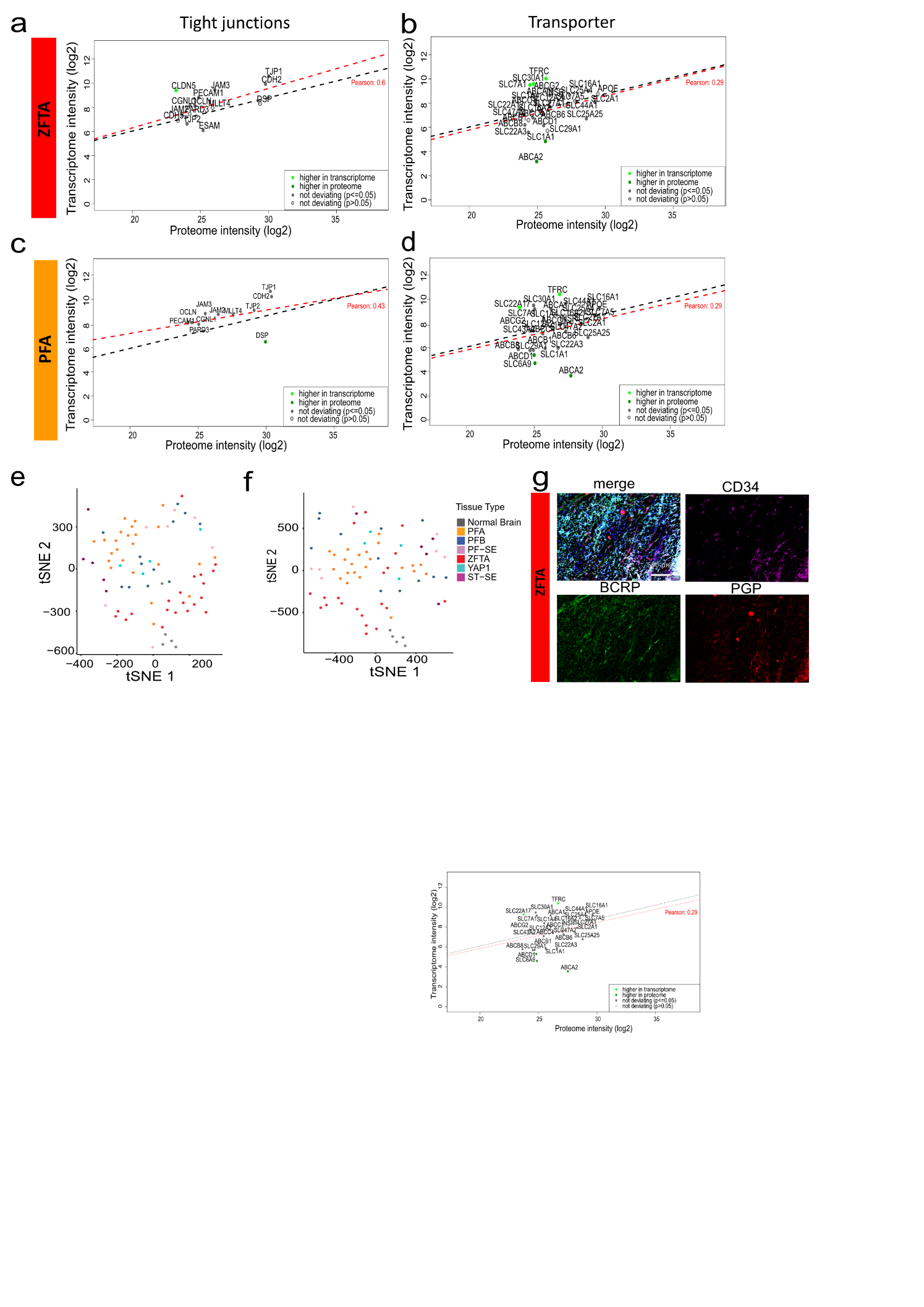


Figure S4: **EPN group-specific RNA-protein correlations. a** Linear regression of tight junction gene RNA vs protein levels in ZFTA. **b** Linear regression of transporter gene RNA vs protein levels in ZFTA. **c** Linear regression of tight junction gene RNA vs protein levels in PFA. **d** Linear regression of transporter gene RNA vs protein in PFA. **e** tSNE plot of EPN protein samples clustered based on tight junctions protein expression and **f** based on transporter and receptor proteins expression. **g** MICS from representative ZFTA and PFA patient tissue, showing tumor cells (CD56,cyan) and endothelial cells (CD34,pink), along with the BCRP (green) and PGP (red).


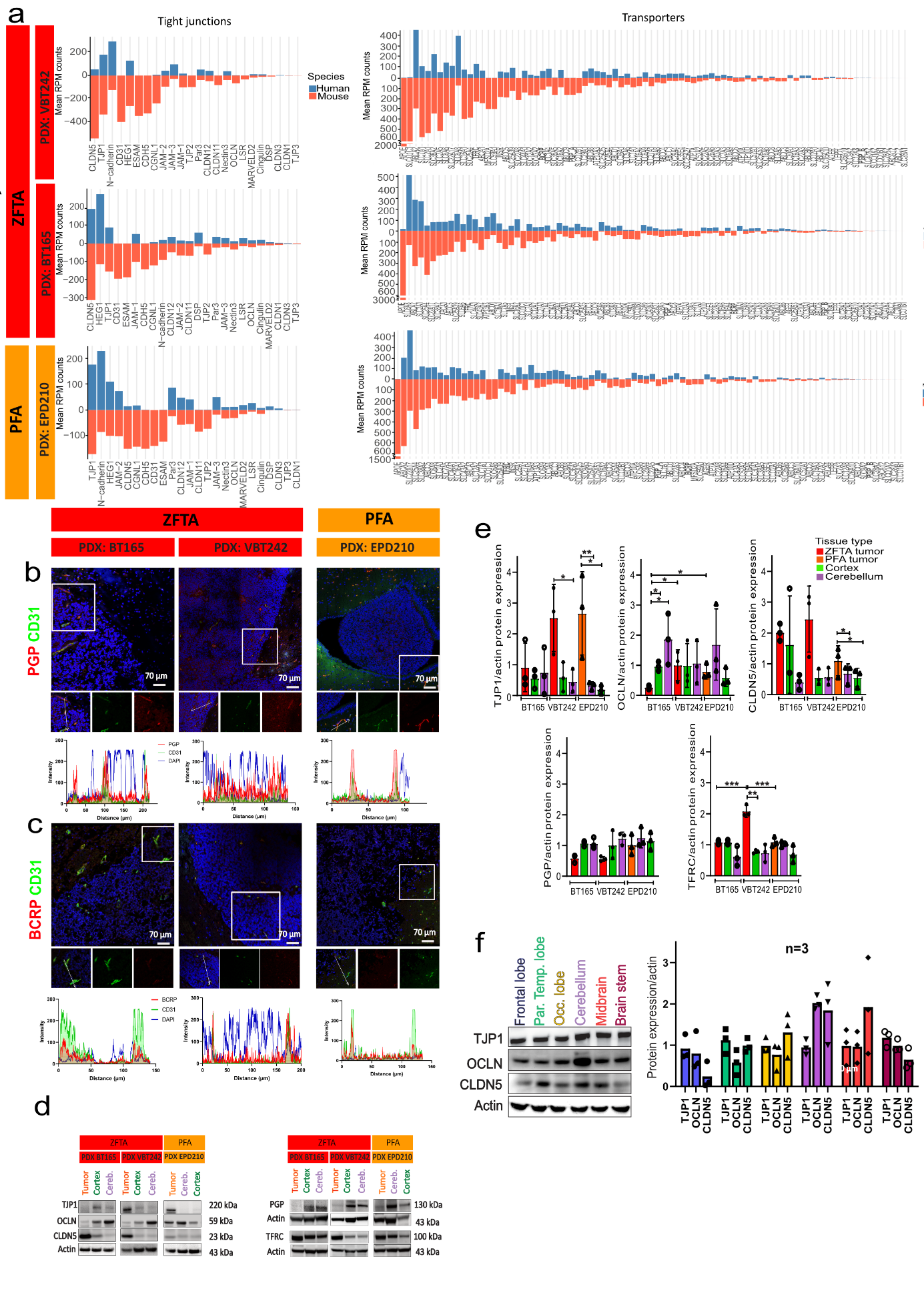


Figure S5: **BBB protein localization in PDX and IUE mouse models. a** Murine and human fraction of BBB genes in PDX models. **b** Immunofluorescence co-staining of CD31 and PGP; **c** and BCRP with CD31. **d** *Representative western blots for TJP1, OCLN, CLDN5, PGP, TFRC, and actin (loading control) in tumor bearing PDX mice and corresponding extra-tumoral brain regions. The original blots are shown in supplementary Figure S7* **e** *Quantification of western blots. Repeated-measure`s one-way ANOVA (within models) and ordinary one-way ANOVA (between tumors) were performed. Only significant differences are indicated as *P < 0.05; **P < 0.0­­­1, ***P < 0.00­­­1* **f** Representative Western Blot of CLDN5 TJP1 and OCLN in tumnor-naive NSG mice and quantitative analysis using actin as loading control (n=3).


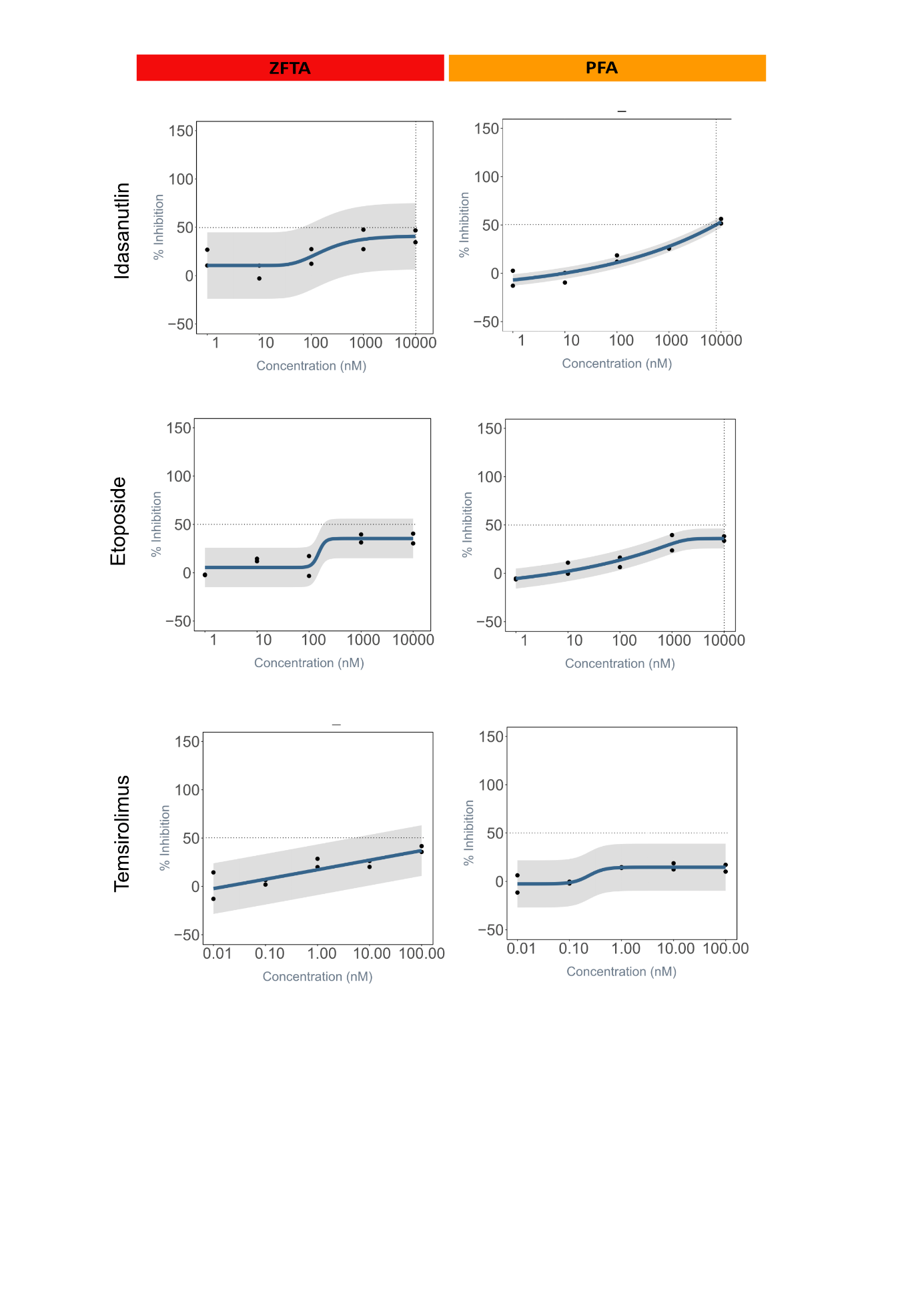


Figure S6: **IC50 curves.** *In Vitro* Inhibition of ZFTA (BT165) and PFA (EPD210) cells by Idasanutlin, Etoposide, and Temsirolimus


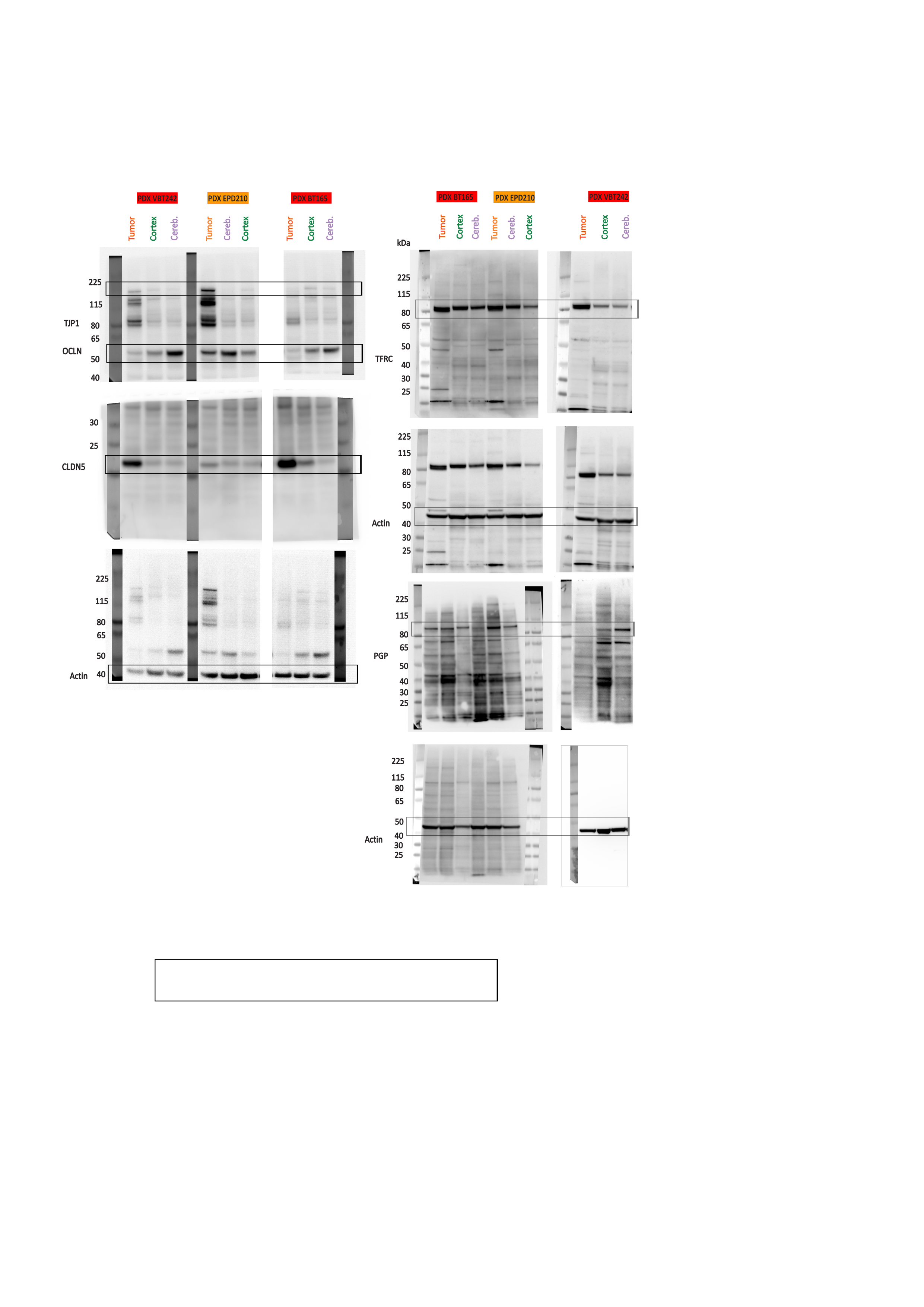
Figure S7: **Original Western blots run on 4-12% Bis-Tris gel.** Membrane was cut, upper part was imaged with OCLN antibody first and afterwards with TJP1 and actin antibody. The lower part was imaged with CLDN5 antibody. TFRC and PGP were detected on separate membranes.
